# Historical data provide new insights into inheritance of traits important for diploid potato breeding

**DOI:** 10.1007/s00425-025-04618-z

**Published:** 2025-02-27

**Authors:** Jadwiga Śliwka, Iwona Wasilewicz-Flis, Henryka Jakuczun, Marta Janiszewska, Paulina Smyda-Dajmund, Karen McLean, Ewa Zimnoch-Guzowska, Glenn J. Bryan, Sanjeev Kumar Sharma

**Affiliations:** 1 Plant Breeding and Acclimatization Institute - National Research Institute in Radzików, Młochów Division, Platanowa St. 19, 05-831 Młochów, Poland; 2 Cell and Molecular Sciences, The James Hutton Institute, Invergowrie, Dundee, DD2 5DA UK

**Keywords:** Boiled tuber taste, Genome-wide association studies, Genotyping-by-sequencing, Next-generation sequencing, Pollen fertility, *Solanum tuberosum*

## Abstract

**Key message:**

Using a diploid potato diversity panel of 246 breeding lines, a genotyping-by-sequencing and a GWAS approach, we mapped QTL for ten traits important to potato breeders, including two previously unmapped traits: boiled tuber taste and pollen fertility.

**Abstract:**

Potato breeding at the diploid level has a long history and has gained new impetus recently, when F1 hybrid breeding was made possible with the discovery of a dominant gene for self-compatibility. Our study deploys a unique diploid diversity panel with a broadened cultivated potato gene pool obtained as a result of introgressing valuable traits from wild potato relatives into the *Solanum tuberosum* background. Using historical phenotyping data collected between 1979 and 2017 for 246 diploid potato clones and high-density genotyping-by-sequencing, we mapped quantitative trait loci (QTL) for tuber yield, mean tuber weight, tuber shape and regularity, tuber eye depth, purple tuber skin colour, flesh colour, tuber starch content, boiled tuber taste (flavour) and pollen fertility. We found some QTL located in genomic regions described in earlier studies, e.g. the QTL for the tuber flesh colour on chromosome 3 overlapping with the location of *beta-carotene hydroxylase* gene. We identified novel QTL for mean tuber weight on chromosomes 8, 9 and 11 and for purple tuber skin colour on chromosomes 6, 7 and 8. QTL for boiled tuber taste and pollen fertility estimated by Lactofuchsin staining have not been mapped before. We found two regions on chromosome 10 affecting the boiled tuber taste, and QTL on chromosomes 2, 4, 5, 6, 9, and 12 for pollen fertility. Considering the increased interest in diploid hybrid potato breeding, the results presented here hold greater relevance and provide novel targets for potato breeding and research at the diploid level.

**Supplementary Information:**

The online version contains supplementary material available at 10.1007/s00425-025-04618-z.

## Introduction

Potato (*Solanum tuberosum* L.) is the fourth most important food crop in the world, after maize, rice and wheat. In 2022, ca. 375 million tonnes of potato were harvested from over 18 million ha worldwide (FAOSTAT 2024). Most cultivated potatoes are tetraploid, the exceptions are diploid landraces and cultivars of the Phureja and Stenotomum Groups grown in the Andes and some long-day-adapted Phureja/Stenotomum cultivars registered in Scotland such as Mayan Gold, Inca Sun, Inca Dawn, Mayan Queen, Mayan Star and Mayan Twilight (Bradshaw 2022). In contrast, almost 75% of the potato wild relatives are diploid (Bethke et al. 2017). Wild potato species originate from a wide range of habitats distributed from southwestern USA to central Chile and Argentina. They are highly diversified and are adapted to a range of harsh environments, which makes them a rich source of traits that cannot be found in the cultivated potato gene pool. For many years, potato breeders have exploited wild germplasm by introgressions into *S. tuberosum* at the diploid level to improve disease resistance and tuber quality, and then the derived germplasm has been used to resynthesise tetraploids for cultivar development (Bethke et al. 2017). This strategy, proposed by Chase (1963) as analytical breeding, was supported by the relative ease of obtaining diploid *S. tuberosum* plants, called dihaploids, from the tetraploid ones and vice versa. To reduce the ploidy, a technique based on induced parthenogenesis has been available since 1960s, with selected Phureja Group pollinators used as inducers of this phenomenon (Bradshaw 2022). To increase the ploidy to tetraploid level, interploidy crosses (4*x* × 2*x*) have been performed, using usually the pollen parent’s ability to produce 2n gametes by first or second division restitution (Zimnoch-Guzowska and Flis 2021). In a diploid potato breeding program in Poland, large pollen grains corresponding to the 2n gametes occurred with different frequencies among the breeding lines (Strzelczyk-Żyta et al. 1997). This breeding strategy not only allowed a widening of the cultivated potato gene pool, but also enabled more efficient genetic studies benefitting from disomic inheritance and aiming at identification of genes and genomic regions affecting various traits. Additional advantage of diploid over tetraploid pre-breeding was the higher probability of obtaining parental lines homozygous for desired alleles and producing non-segregating progenies (Zimnoch-Guzowska and Flis 2021). Final steps of cultivar breeding faced the challenges of tetrasomic inheritance of multiple alleles, inbreeding depression, and the limited rate of tuber reproduction (Clot et al. 2020). The solution could be the next development in potato breeding, F1 hybrid breeding at diploid level which was made possible with the discovery of a dominant gene for self-compatibility *Sli* (*S*-locus inhibitor, Hosaka and Hanneman 1998) and better understanding of self-compatibility of diploid potatoes (Clot et al. 2020; Bradshaw 2022).

A diploid parental line breeding program started at Plant Breeding and Acclimatization Institute—National Research Institute (IHAR-PIB), Młochów Division, Poland in 1968 (Zimnoch-Guzowska and Flis 2021). The diploid potatoes were used as sources of resistance to various diseases and quality traits, such as high tuber starch content, cooking or chipping quality, and resistance to potato leaf roll virus, potato virus M, *Synchytrium endobioticum*, *Phytophthora infestans* and pectinolytic bacteria. Through crosses at the diploid level, valuable traits from wild potato relatives and the cultivated Andean potato species were introgressed into an *S. tuberosum* genetic background, and with use of *2*n gametes they widened the genetic pool of tetraploid cultivars. In 1997, ca. 70% of tetraploid parental lines developed at IHAR-PIB originated from the diploid parental lines. In turn, the tetraploid lines from IHAR-PIB became parents of 72 potato cultivars registered in Poland between 1985 and 2019. The main goals of the diploid breeding program in Poland have changed since its beginning, evolving from breeding high-yielding potatoes for general use or starch production, to potatoes of high table quality and suitable for specialised processing. Emphasis on breeding for resistance to viruses shifted to resistance to late blight and pectinolytic bacteria. In the selection process in total ca. 30 characters were evaluated but changed over time to correspond with the breeding goals (Zimnoch-Guzowska and Flis 2021).

In this study, we focused on those traits that were continuously important and evaluated in diploid potato pre-breeding programs at IHAR-PIB. Using historical phenotyping data collected between 1979 and 2017 for 246 diploid potato clones and high-density genotyping-by-sequencing, we mapped QTL for yield, mean tuber weight, tuber morphology traits, starch content, boiled tuber taste, and pollen fertility.

## Materials and methods

### Phenotyping

Plant material comprised 246 diploid potato breeding lines (Supplementary Table S1) that are maintained at IHAR-PIB, as a part of the diploid potato collection (POL047), hereafter referred to as ‘diploid diversity panel’ (DDP). The 228 diploid potato clones were bred at IHAR-PIB Młochów (Poland) in the years 1979–2012, with the remaining 18 clones coming from the Danish, German, Dutch, Italian and Russian potato collections. The clones were obtained by recombinant breeding in several breeding directions listed in Table 1 along with the numbers of lines from each breeding program included in DDP, and the *Solanum* spp. introgressed in breeding programs. Main breeding aim for each potato line is given in Supplementary Table S1. In addition to the main breeding goals, traits such as yield, mean tuber weight, tuber shape, regularity of tuber shape, tuber eye depth, tuber flesh colour, and tuber starch content were evaluated for all 246 clones. Pollen staining, tuber skin colour, and the boiled tuber taste were assessed for 240, 217, and 96 clones, respectively.

**Table 1:** Composition of the diploid diversity panel. *Solanum tuberosum* dihaploids were used in all breeding programs and are present in pedigrees of 240 out of 246 lines in the panel

Breeding program (main aim)	Number of potato lines	Introgressed *Solanum* spp.
High starch content	40	*S. chacoense*,* S. verrucosum, S.* *yungasense*
Good table quality and taste	48	*S. goniocalyx, S. phureja*
Chipping quality	45	*S. goniocalyx, S. phureja*
Resistance to potato viruses	34	*S. acaule*^a^, *S. chacoense*, *S. gourlayi*, *S. megistacrolobum, S. phureja*, *S. stoloniferum*^a^, S. *tuberosum* subsp. *andigena*^a^, *S.* *yungasense*
Resistance to *P. infestans*	60	*S. michoacanum, S. microdontum*, *S. phureja*, S. *pinnatisectum, S. ruiz-ceballosii*, *S. stenotomum*, *S. verrucosum*
Resistance to soft rot and blackleg	8	*S. chacoense*, *S. phureja, S.* *yungasense*
Ability for 2*n* gametes formation	8	*S. chacoense*, *S. phureja*
Resistance to nematodes	3	*S. gourlayi*, *S. tuberosum* subsp. *andigena*^a^, *S.* *vernei*

^a^Species in the pedigree of tetraploid potato cultivars or breeding lines that were used for obtaining dihaploids

The plants of each clone were grown in the field in Młochów Division of IHAR-PIB, Poland, between 1979 and 2017 in a single plot (with some exceptions planted in 2 or 3 plots) with 7 plants per plot (exceptions with 15 plants). Particular clones were grown and evaluated in varying number of years between 2 and 37. The field experiments were fertilized with 90, 90, and 170 kg/ha of N, P, and K, respectively, and potatoes were chemically protected against pests and pathogens, according to the standard recommendation in particular years of the period 1979–2017. The best linear unbiased estimates (BLUEs) for all traits, except tuber skin colour, were calculated using REML implemented in Genstat 20th edition (VSN International Limited, http://www.vsni.co.uk). Tuber skin colour was scored on a nominal scale, and data were curated manually and assigned into the respective categories described in the section below. During the assessment of all traits, the registered tetraploid cultivars were used as standards with defined catalogue values of the traits in order to relate the performance of the diploid clones to the elite material.

This study reports findings for the following traits:

- Yield (YLD, g/plant);
- Mean tuber weight (MTW, g);
- Tuber shape [SHP; 1–6 scale according to Domański (2001a): 1 = compressed (length/width ratio < 0.9), 2 = round, 3 = round-oval, 4 = oval, 5 = long-oval, and 6 = long (length/width ratio > 2.0)];
- Regularity of tuber shape [REG; 1–9 scale as described by Hara-Skrzypiec et al. (2018a) after Domański (2001a): 1 = highly malformed tubers and 9 = almost all tubers of the same shape];
- Tuber eye depth [EYE; 1–9 scale according to Domański 2001a: 1 = eyes deeper than 5 mm, 9 = eyes impalpable];
- Tuber skin colour [a–e scale according to modified descriptors of Huamán et al. (1977): a = white, b = pink, c = purple, d = brown and e = purplish white]. GWAS was performed for purple tuber skin colour (PUR) after transforming the data to binary scores i.e. purple (purple, purplish white) versus non-purple (white, brown) while the pink skin colour (one clone only) was excluded from the analysis.
- Tuber flesh colour [TFC; 1–6 scale according to Śliwka et al. (2008): 1 = white, 2 = greyish white, 3 = creamy white, 4 = pale yellow, 5 = yellow, 6 = deep yellow flesh colour] evaluated on five tubers per clone cut along shorter axes;
- Tuber starch content (TSC, %) calculated by the underwater weight method, on the basis of the ratio of tuber weight in the air to tuber weight in water, according to Lunden (1956);
- Boiled tuber taste [TST; 1–9 scale as described by Anonymous (1974) and Domański (2001b): 1 = very bad taste, 2 = bad taste, 3 = severe flaws of taste or smell, potato not suitable for consumption, 4 = mild flaws of taste or smell, 5 = lack of distinctive taste, 6 = medium taste, 7 = satisfactory good taste, 8 = good taste, 9 = very good taste] assessed by 3–5 panellists using six peeled potato tubers cooked in unsalted water until soft;
- Pollen fertility (POL, % of Lactofuchsin-stained grains) estimated by an indirect method of staining with Lactofuchsin: 20 ml of phenol, 20 ml of lactic acid, 40 ml of glycerine and 8 ml of 1% solution of fuchsin in water (Abdalla 1970; Wasilewicz-Flis and Jakuczun 2001). Pollen was collected from three flowers per plant, approximately 24 h after anthesis and between 10 am and 3 pm when, according to Abdalla (1970), the results are most reliable. Immediately after adding Lactofuchsin to the pollen, percentage of round and deeply stained pollen grains was counted under a microscope (Janssen and Hermsen 1976). Pollen with at least 30% of grains stained was considered fertile and the threshold was applied as a selection criterion in breeding (Wasilewicz-Flis and Jakuczun 2001).

### Genotyping-by-sequencing (GBS)

Genomic DNA from 200 mg of fresh young leaf tissue was extracted from individual plants using DNeasy Plant Mini Kit (Qiagen, Hilden, Germany). DNA was quantified using the Quant-iTTM PicoGreen^®^ dsDNA Assay Kit (Invitrogen, San Diego, CA, USA). For the construction of GBS libraries, DNA samples (100 ng each) from the association panel were processed through a double (*Pst*I-*Mse*I) restriction enzyme digestion followed by adapter ligation. Each sample was uniquely barcoded using *Pst*I adapters (Supplementary Table S2) whereas a single *Mse*I adapter was used as common adapter for all samples. All processed samples per each GBS library were pooled together, PCR-amplified and size-selected (300 bp–500 bp) using BluePippin™ (Sage Science Inc., Beverly, MA, USA). Fragment size assessment and further quality checks on pooled GBS library samples were performed using Bioanalyzer High-Sensitivity DNA chip (Agilent Technologies, Santa Clara, CA, USA). The sequencing was performed on Illumina HiSeq 2500 platform to generate 150 bp paired-end sequence reads keeping one GBS library per lane. GBS was performed using *Pst*I/*Mse*I restriction enzyme combination as reported for potato by Sharma et al. (2024), adapted from the original dual restriction enzyme digestion procedure described by Poland et al. (2012), keeping a 96-plex (i.e. 96 samples pooled per GBS library) format.

### Variant discovery and genotype calling

Sequence read data for each GBS library were demultiplexed using GBS-SNP-CROP-v.4.1 (Melo et al. 2016), quality trimmed using Trimmomatic (Bolger et al. 2014) and mapped onto the DM (doubled monoploid potato *S. tuberosum* Group Phureja DM 1–3 516 R44) potato reference genome version 6.1 (Potato Genome Sequencing Consortium 2011; Pham et al. 2020) using Bowtie2 (Langmead and Salzberg 2012). Variant discovery was done using HaplotypeCaller followed by joint genotyping across all panel samples using GenotypeGVCF tools available through GATK (McKenna et al. 2010; DePristo et al. 2011). The raw variants were filtered for read depth (DP >  = 10), genotype quality (GQ >  = 10) and QualByDepth (QD >  = 2) using GATK VariantFiltration and SelectVariants tools. For genetic analysis, SNPs displaying higher (> 20%) missing data, lower (< 1%) minor allele frequency (MAF) and higher (> 0.98) maximum genotype frequency (highest frequency of a specific genotype in the panel after applying dominance relations) were filtered out.

### Population structure and genome-wide association analysis

Population stratification in the panel was assessed using the principal component analysis (PCA) performed over genomic relationship matrix implemented in the R package ASRgenomics. The number of subpopulations (Q) in the association panel was inferred from PCA scree plot. The genetic kinship among the panel genotypes was visualized using a dendrogram heatmap of the genomic relationship matrix. GWAS was performed using GWASpoly version 2.10 (Rosyara et al. 2016) employing additive, general and simplex dominance genetic effect models as described in GWASpoly manual. Each genetic effect model was further examined using four different statistical models namely, (i) Naïve model, without controlling any confounding effects, (ii) Kinship model, factoring for familial relatedness only (K), (iii) Population Structure model, controlling for population structure (Q) effects only, and (iv) Full model, adjusting both K as well as Q population confounding effects; hereafter referred to as Naïve, K, Q and QK models, respectively. Fitness of four statistical models (viz., Naïve, K, Q and QK) within each genetic effect model was assessed using Quantile–Quantile (Q-Q) plots of the expected versus observed − log_10_(*p*) values, and models were ranked using genomic control inflation factor (λ_GC_) metric which was calculated as the median of the resulting chi-squared test statistics divided by the expected median of the chi-squared distribution. The genome-wide *P*-value (− log_10_(*P*)) detection threshold for statistical significance was calculated using the LD-based Bonferroni-type multiple testing correction method “M.eff” (genome-wide α = 0.05).

### Linkage disequilibrium decay

Linkage disequilibrium (LD), at whole chromosome-scale level, was calculated following the procedure as previously described (Vos et al. 2017; Sharma et al. 2018). The correlations (Pearson correlation coefficient, *r*^*2*^) between marker-pairs were calculated using SNP dosage scores which were further used to derive LD estimates for all 12 individual chromosomes employing marker pairs located within each respective chromosome. The extent of LD decay was computed by implementing Quantile regression (R package 'quantreg'; Koenker 2017) on the 90th percentile. From the regression analysis, the estimates of LD_1/10,90_, indicating the genomic distance (in Mb) at which LD equals one-tenth of its maximum fitted *r*^*2*^ value (*r*^*2*^max) on the set percentile, were obtained for all 12 potato chromosomes.

The bioinformatics and computational analyses were performed on Crop Diversity High-Performance Compute (HPC) Cluster, described by Percival-Alwyn et al. (2024).

## Results

### Phenotyping

**Fig. 1:**
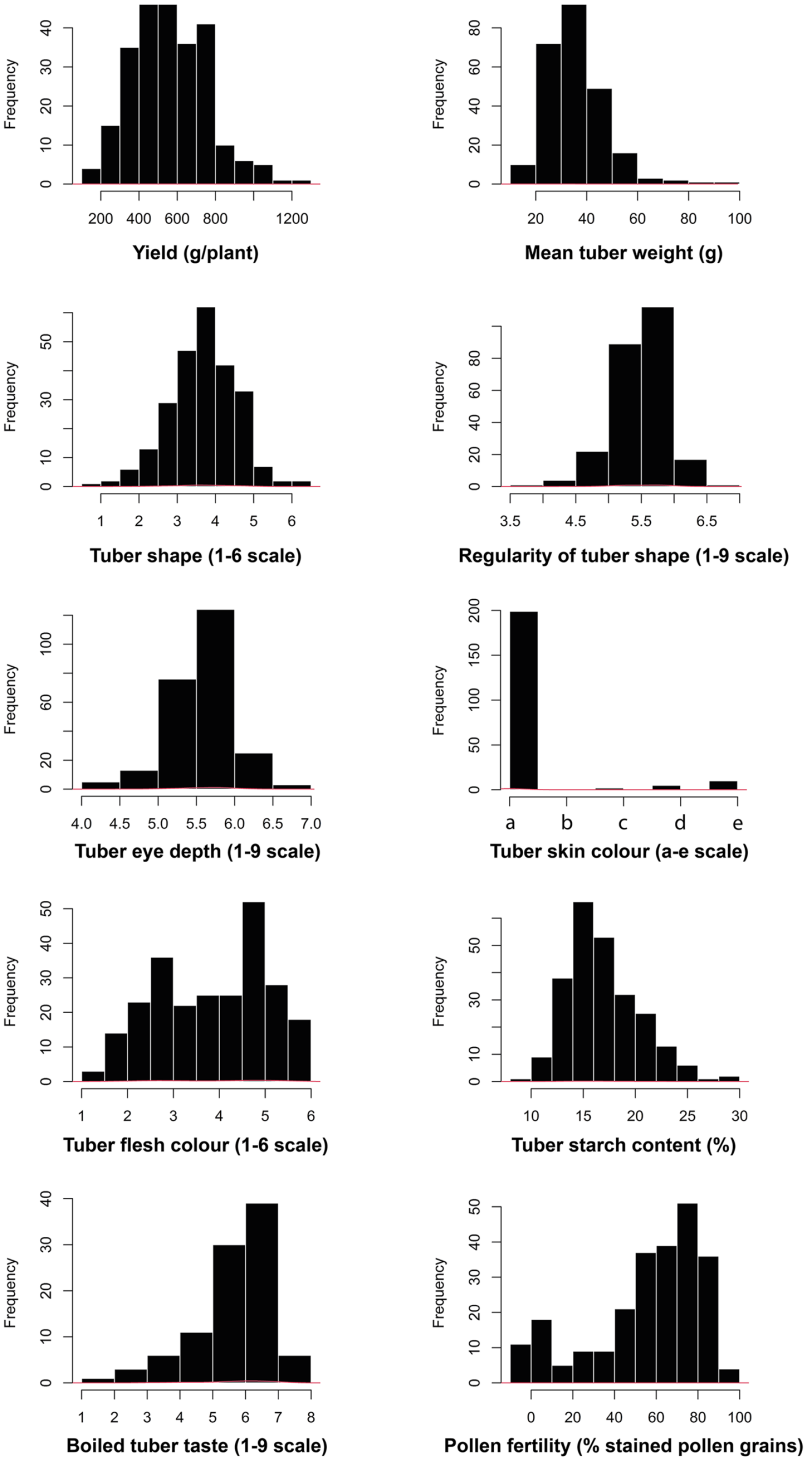
The distribution of individual trait values

The distributions of the ten analysed traits in the diploid diversity panel are shown in Fig. 1. BLUEs of the YLD for potato genotypes varied between 135.3 and 1280.1 g/per plant while the MTW range was 12.8–90.7 g. Although SHP displayed all tuber shapes from compressed (score 1) to long (score 6), the propensity for potato genotypes with round-oval and oval tubers (scores 3 and 4) was higher. Genotypes with either extremely irregular (score 1) or very regular tubers (score 9) were absent in the panel resulting in narrowing the range of BLUEs of REG scores to 3.6–6.6. The range of EYE noted in the panel was 4.1–6.7, also not covering the full 1–9 scale used for assessment of this trait. Regarding tuber skin colour, tubers of all five skin colours were present in the panel but genotypes with white skins dominated. Distribution of TFC displayed a wide range over the scored categories with overrepresentation of genotypes classified as having either greyish to creamy white (scores 2.5–3) or pale yellow to yellow tuber flesh (scores 4.5–5). BLUEs of the TSC ranged between 8.6 and 29.2%. Most genotypes within the diploid diversity panel were scored between 5 and 7 for the TST. The distribution of POL showed signs of selection for this trait, with the genotypes with pollen staining of 30% and less being underrepresented in the panel (Fig. 1).

**Fig. 2:**
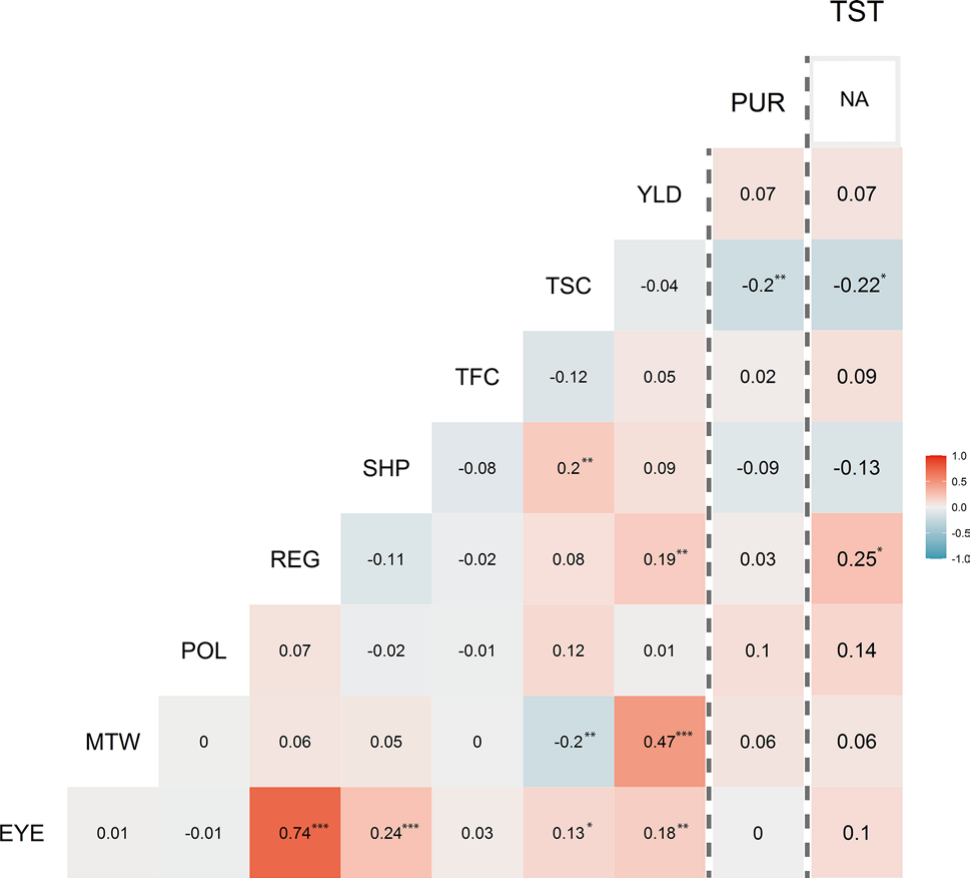
Correlation heatmap of all 10 traits included in the study: *YLD*, Yield; *MTW*, Mean tuber weight; *SHP*, Tuber shape; *REG*, Regularity of tuber shape; *EYE*, Tuber eye depth; *PUR*, Purple tuber skin colour; *TFC*, Tuber flesh colour; *TSC*, Tuber starch content; *TST*, Boiled tuber taste; *POL*, Pollen fertility. The vertical dashed lines are to emphasize that correlation values for TST and PUR are using 92 and 210 samples, respectively, while the sample-size for the rest of the traits is 240. NA = No variation for PUR in the 92 clones evaluated against TST. Correlations significant at: *P < 0.05, **P < 0.01, ***P < 0.001

Significant correlations were observed between some traits while correlations between most trait-pair combinations were non-significant (Fig. 2). The shallow eyes were more often found in regular and elongated tubers, as the strongest correlation among all evaluated traits was detected between EYE and REG, and EYE was also significantly correlated with SHP. The TSC was positively correlated with SHP and EYE and negatively correlated with MTW, PUR, and TST. YLD was positively correlated with MTW, REG, and EYE. TST was assessed in a subset (*n* = 96) of genotypes and the trait showed significant correlation with TSC and REG.

### Genotyping-by-sequencing

**Fig. 3:**
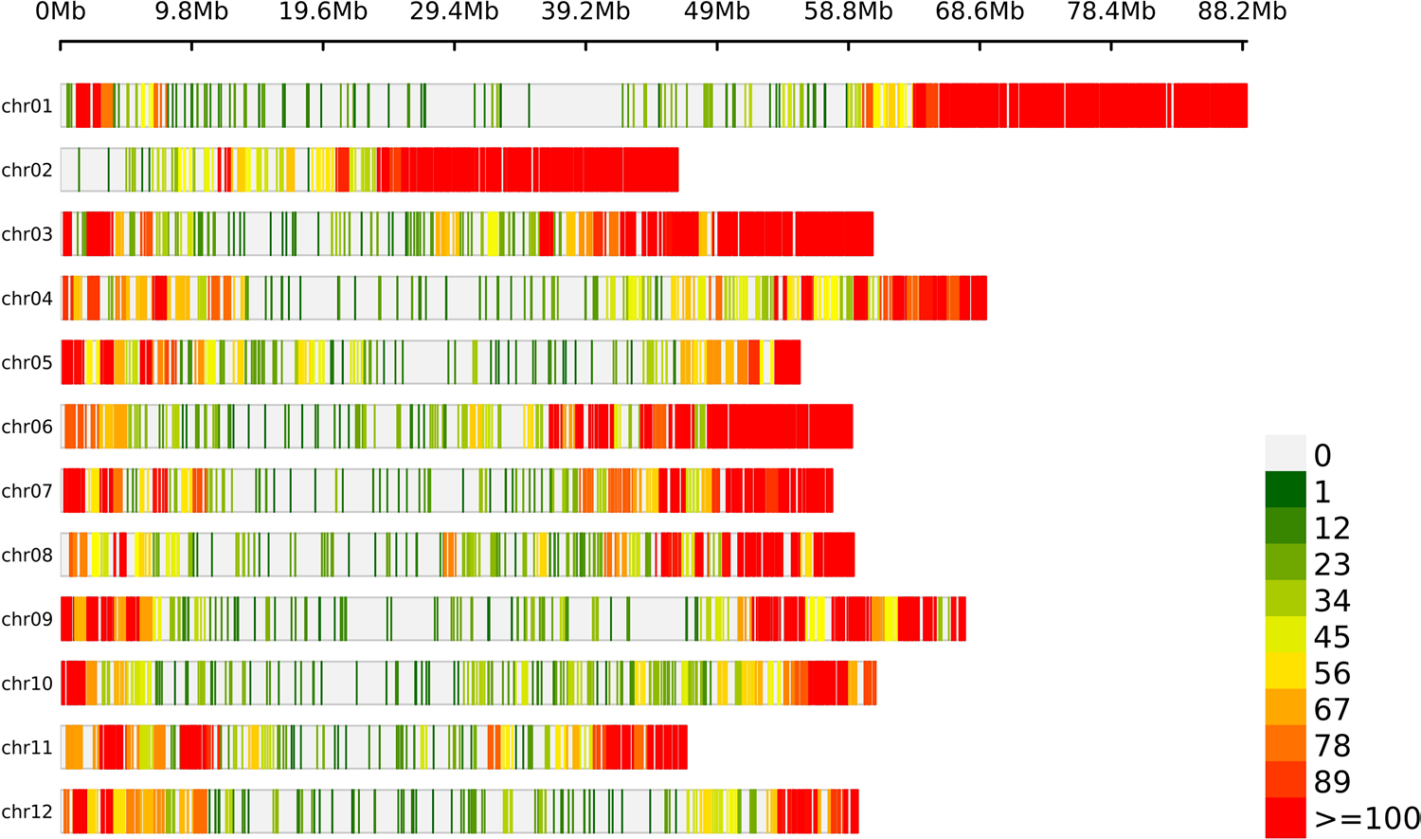
Distribution of 39,756 GBS SNPs used in all genetic analyses. Bin size = 1 Mb

Genotyping-by-sequencing was performed using dual restriction enzymes (*Pst*I/*Mse*I) digestion following the procedure described by Sharma et al. (2024). Applying read mapping and variant discovery procedure as described in the Materials and methods section followed by variant filtering for sample genotype level read depth (DP < 10), low genotype quality (GQ < 10), quality-by-depth (QD < 2.0) and excluding non-SNP and monomorphic variants yielded 187,708 high quality SNPs indicating an average of one GBS SNP per ~ 3.95 kb. Of these, 99.9% SNPs (187,574) were physically mapped across 12 potato chromosomes. The highest and lowest marker rates were observed on chromosome 10 and chromosome 1 with an average of one SNP per ~ 6.2 kb and ~ 13 kb, respectively. The remaining 134 SNPs were physically mapped on unanchored superscaffolds (chromosome 0) of the potato genome. The genomic locations of the identified GBS SNPs intersected 20,803 genes (Supplementary Table S3). Figure 3 displays genome-wide SNP density plot and the distribution of SNPs by chromosomes is given in Table 2. The panel showed a Ts/Tv (Transitions/Transversions) ratio of 1.86 for the observed SNPs while the missense-to-silent ratio for the panel SNPs was 0.88 (missense: 46.54%; silent: 53.08%; nonsense: 0.39%). The percentage of SNPs with high, low, moderate, and modifier impact categories was 0.15, 13.10, 10.58, and 76.18, respectively. The number of SNPs in different effect-type categories and regions is provided in Table 3. SNPs were further processed for removing variants displaying higher (> 20%) missing data, lower (< 1%) minor allele frequency (MAF) and higher (> 0.98) maximum genotype frequency, yielding a robust set of 39,756 SNPs used for all subsequent analyses. The genotypes were called in allele dosage format (0, 1, 2) using R package GWASpoly (Rosyara et al. 2016).

**Table 2:** GBS SNP variant rate (average genomic distance per SNP) per chromosome

Chromosome	Length (bp)	SNP count	SNP rate (bp)
1	88 591 686	24 400	3 630
2	46 102 915	19 251	2 394
3	60 707 570	17 610	3 447
4	69 236 331	16 804	4 120
5	55 599 697	12 163	4 571
6	59 091 578	17 048	3 466
7	57 639 317	14 143	4 075
8	59 226 000	14 043	4 217
9	67 600 300	14 501	4 661
10	61 044 151	11 659	5 235
11	46 777 387	13 935	3 356
12	59 670 755	12 017	4 965
0	10 297 348	134	76 845
Total	741 585 035	187 708	3 950

**Table 3:** Details of GBS SNP effects by type and region

Effect type	SNP Count	Percent^a^
3_prime_UTR_variant	7 296	2.21%
5_prime_UTR_premature_start_codon_gain_variant	291	0.09%
5_prime_UTR_variant	2 092	0.64%
Downstream_gene_variant	84 255	25.56%
Start_codon_variant	3	0.00%
Intergenic_region	35 846	10.88%
Intron_variant	68 979	20.93%
Missense_variant	34 445	10.45%
Splice_acceptor_variant	72	0.02%
Splice_donor_variant	62	0.02%
Splice_region_variant	3 864	1.17%
Start_lost	21	0.01%
Stop_gained	286	0.09%
Stop_lost	48	0.02%
Stop_retained_variant	50	0.02%
Synonymous_variant	39 317	11.93%
Upstream_gene_variant	52 694	15.99%

Effect Region	SNP Count	Percent^*b*^
DOWNSTREAM	84 255	25.87%
EXON	73 688	22.63%
INTERGENIC	35 846	11.01%
INTRON	65 903	20.24%
SPLICE_SITE_ACCEPTOR	72	0.02%
SPLICE_SITE_DONOR	62	0.02%
SPLICE_SITE_REGION	3 476	1.07%
UPSTREAM	52 694	16.18%
UTR_3_PRIME	7 296	2.24%
UTR_5_PRIME	2 383	0.73%

^a^Percent SNPs out of the total SNP count for all 'Effect Type' categories

^b^Percent SNPs out of the total SNP count for all 'Effect Region' categories

### Linkage disequilibrium decay

**Fig. 4:**
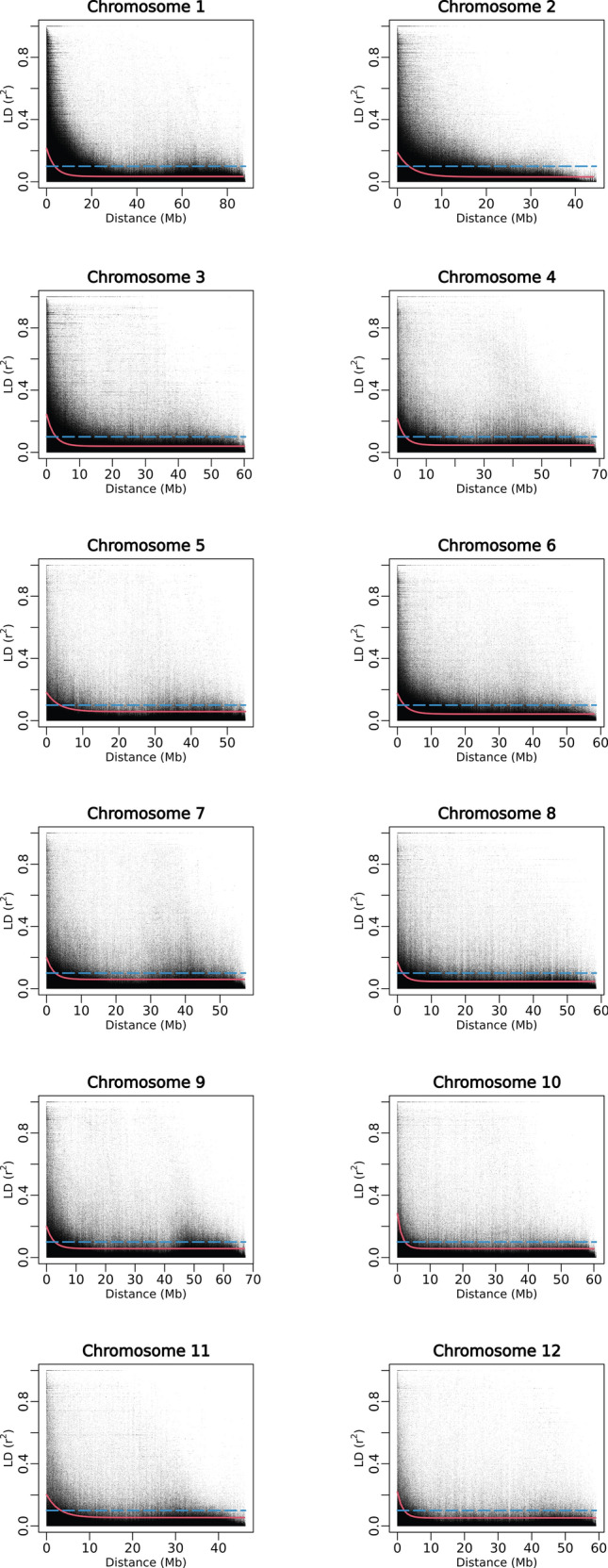
Linkage disequilibrium (LD) measure *r*^*2*^ in DDP plotted versus the physical map distance (Mb) between pairs of 39,756 GBS SNPs located on the whole chromosomal region for all 12 chromosomes. The trend line of the nonlinear quantile regression of *r*^*2*^ (90th percentile) versus the physical map distance between the SNP markers is illustrated in red while dashed blue line depicts the standard LD decay threshold (*r*^*2*^ = 0.1)

Linkage disequilibrium in DDP was assessed using Pearson’s *r*^*2*^ statistic using pairwise combinations of SNPs present across all 12 chromosomes. The extent of LD decay was estimated at the whole chromosome level using the LD_1/10,90_ estimator denoting the distance (in Mb) at which LD equals one-tenth of its maximum fitted *r*^*2*^ value (*r*^*2*^_max,90_) on the 90th percentile. The extent of LD decay observed in DDP using 39,756 SNPs is illustrated in Fig. 4 while the *r*^*2*^_max,90_ and LD_1/10,90_ estimates are provided in Table 4. The average LD_1/10,90_ estimate for the panel was 2.67 Mb (Table 4). Relative to all 12 potato chromosomes, LD_1/10,90_ metric revealed faster LD decay rate for chromosome 8 while a slower LD decay rate was observed for chromosome 5.

**Table 4:** Extent of LD decay in DDP estimated using GBS SNPs at whole chromosome level

Chromosome	*r*^*2*^_max,90_^a^	LD_1/10,90_ (Mb)^b^
1	0.21	3.59
2	0.19	2.57
3	0.24	2.83
4	0.22	2.81
5	0.18	3.68
6	0.18	2.07
7	0.20	2.49
8	0.17	1.54
9	0.20	2.76
10	0.28	2.32
11	0.20	3.30
12	0.22	2.01
Average	0.21	2.67

^a^*r*^*2*^_max,90_: Maximum Pearson correlation coefficient (*r*^*2*^) achieved in the 90th percentile

^b^LD_1/10,90_: Physical distance (Mb) at which LD has decayed to one-tenth of its maximum *r*^*2*^ value in the 90th percentile

### Genetic characterization and assessment of population stratification

**Fig. 5:**
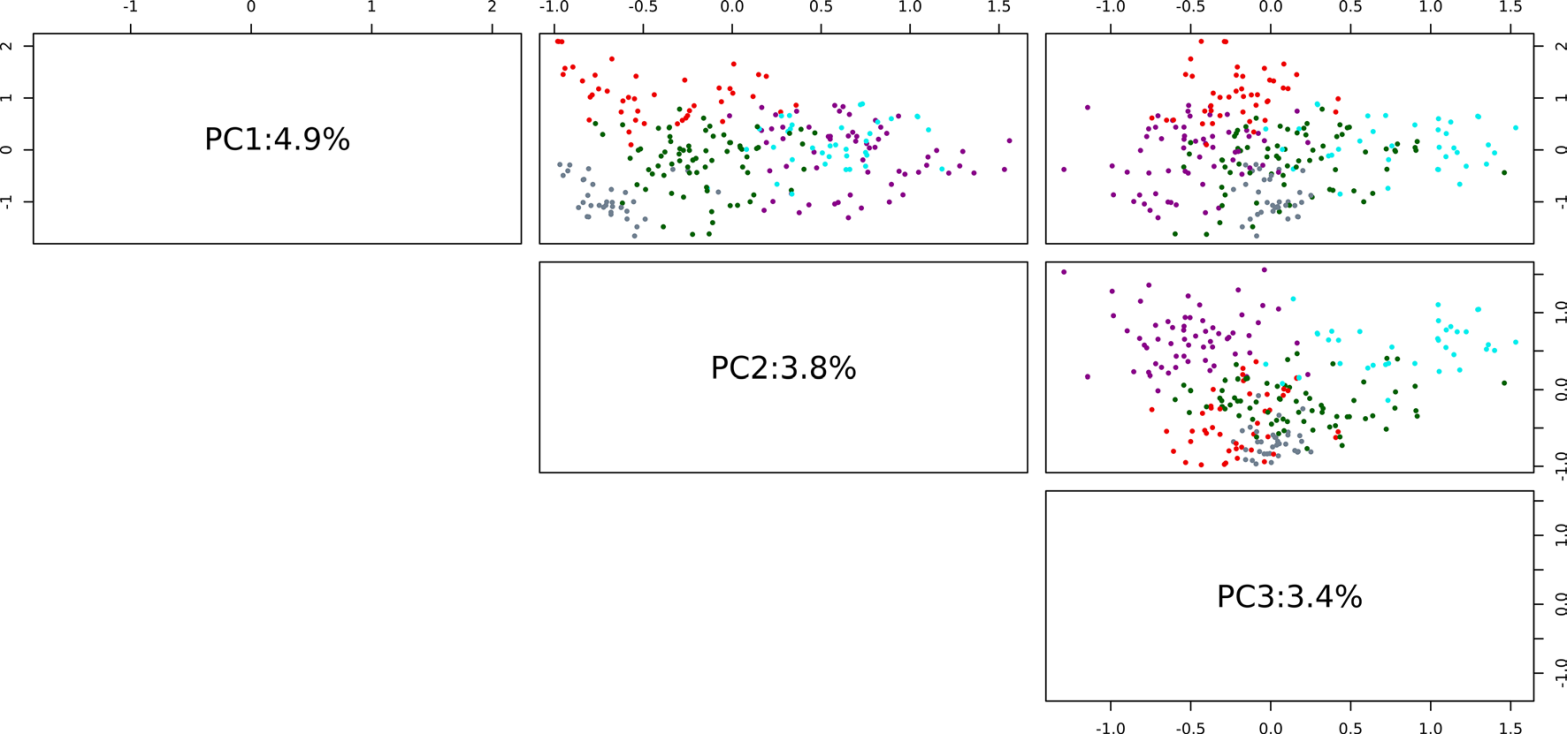
Principal component analysis of 246 DDP clones using 39,756 GBS SNPs. The individual clones are coloured on the basis of their membership to subpopulations (clusters) detailed in Supplementary Table S1 (Cluster1 = red, Cluster2 = green, Cluster3 = cyan, Cluster4 = grey, Cluster5 = magenta)

**Fig. 6:**
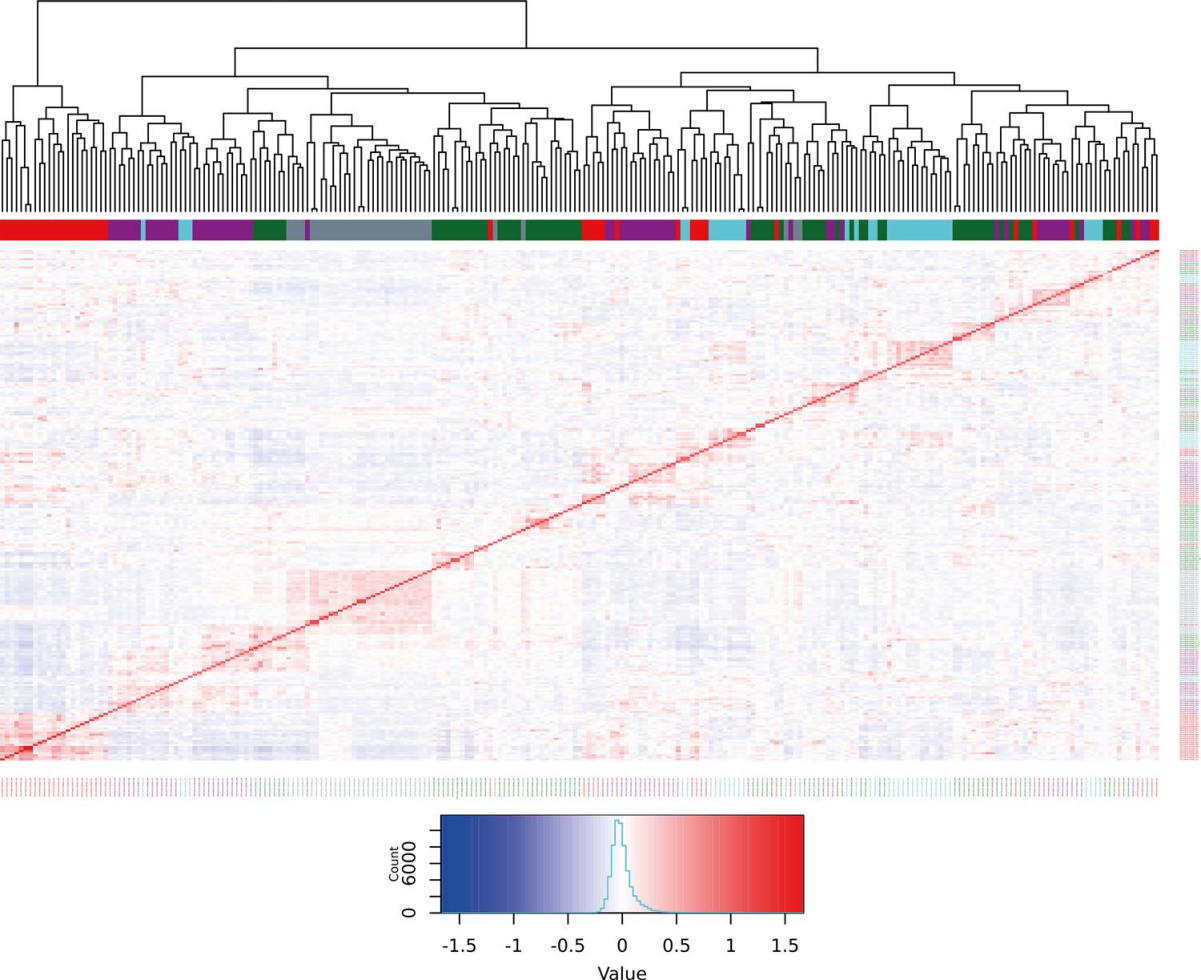
Heatmap displaying relationships among 246 DDP clones using 39,756 GBS SNPs. The red diagonal represents perfect relationship of each line with itself; the symmetric off-diagonal elements represent relationship for pairs of lines where warmer colour indicates a positive relationship and colder colour denotes a negative relationship. The colours in the horizontal bar below dendrogram depict clones’ membership to subpopulations (clusters) as detailed in Supplementary Table S1 (Cluster1 = red, Cluster2 = green, Cluster3 = cyan, Cluster4 = grey, Cluster5 = magenta)

DDP was genetically characterized using 39,756 GBS SNPs discovered in the current study and their pattern of distribution and marker density are illustrated in Fig. 3. The overall distribution of minor allele frequency (MAF), maximum genotype frequency and polymorphism information content (PIC) observed in the panel is shown in Supplementary Fig. S1 while their chromosome-wise distribution is presented in Supplementary Fig. S2. As illustrated in these figures, SNPs displayed a full range for these key marker properties in the DDP with average values (MAF = 0.17, maximum genotype frequency = 0.75, PIC = 0.19) keeping in the moderate informative range typical of SNPs identified through next-generation sequencing-based approaches. The population structure was analysed using principal component analysis (PCA) and K-means clustering. The point of inflection (elbow junction) in the PCA scree plot indicated the presence of five subpopulations (Q) within the association panel (Supplementary Fig. S3). These observations were further corroborated by the cluster detection based on Bayesian information criterion (BIC) which supported the presence of five clusters as ‘k = 5’ was within the shallow minimum of the BIC ‘goodness of fit’ curve (Supplementary Fig. S4) indicating five clusters are most optimum for classifying genotypes in the panel. The pattern of population stratification and the percentage of the genetic variation accounted by the first three components of the PCA is illustrated in Fig. 5. The K-means-based cluster (subpopulations) membership for the DDP clones is provided in Supplementary Table S1. The genetic kinship among the genotypes included in DDP was visualized as a dendrogram heatmap of the genomic relationship matrix (Fig. 6).

### GWAS results

**Fig. 7:**
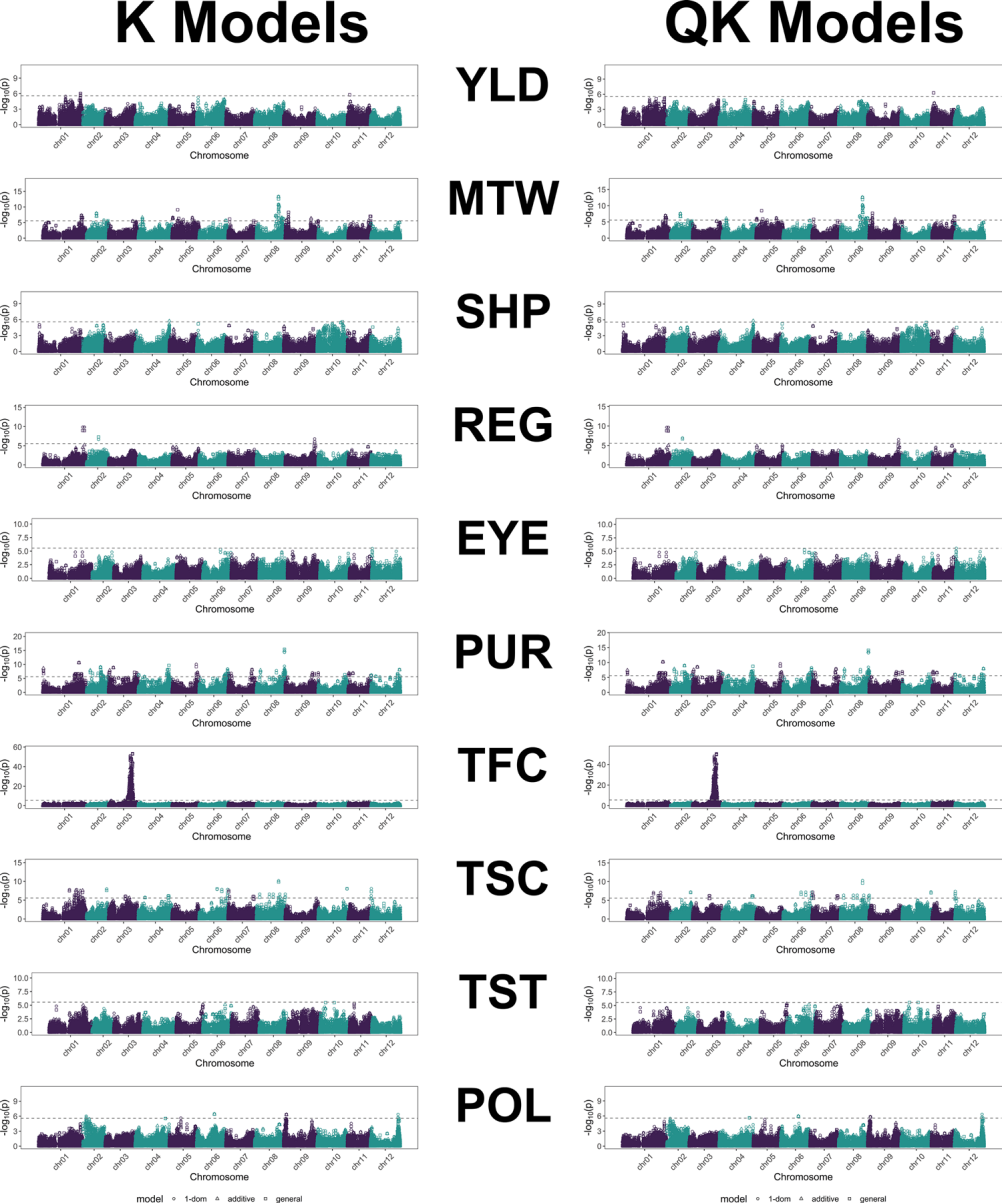
Trait-wise combined Manhattan plots from all genetic (gene action) models performed for each GWAS statistical model using 39,756 GBS SNPs. GWAS significance thresholds (dashed lines), specific to each trait, are derived using Bonferroni-type multiple testing correction method “M.eff” (genome-wide α = 0.05). The GWAS significance threshold varies across different genetic (gene action) models, therefore, only the most stringent value obtained among these models is plotted in the combined Manhattan plots. *YLD*, Yield; *MTW*, Mean tuber weight; *SHP*, Tuber shape; *REG*, Regularity of tuber shape; *EYE*, Tuber eye depth; *PUR*, Purple tuber skin colour; *TFC*, Tuber flesh colour; *TSC*, Tuber starch content; *TST*, Boiled tuber taste; *POL*, Pollen fertility

GWAS for all traits employed additive, general and simplex dominance gene action models as described in the R package GWASpoly manual. Each gene action model was further evaluated using Naïve, K, Q and QK models, and fitness of these statistical models was evaluated using Q-Q plots and genomic control inflation factor (λ_GC_) metric as described in Materials and methods. First five principal components (PC) were deemed relevant for describing population structure based on the point of inflection observed in the PCA scree plot (Supplementary Fig. S3) and BIC 'cluster detection’ goodness of fit curve (Supplementary Fig. S4), and were included to form the Q matrix in Q and QK GWAS models for controlling population confounding effects. The marker-trait associations (MTAs) were declared statistically significant based on *P*-value (− log_10_(*P*)) detection threshold established using the "M.eff" method (implemented in GWASpoly) to control the genome-wide false positive rate (α = 0.05). Supplementary Fig. S5 displays Q-Q plots comparing the inflation of *P*-values for the four principal GWAS models deployed for each genetic (gene action) model for all 10 traits and Supplementary Table S4 presents genomic control inflation factor (λ_GC_) values for each trait and 'GWAS genetic model x statistical model' combination. Figure 7 illustrates individual trait Manhattan plots from all GWAS and gene action models analysed here.

GWAS scans were performed for selecting the most significant marker per LD-based moving window and a single MTA per LD-based window (genomic interval) was retained (hereafter referred to as QTL-MTA) for each gene action model GWAS. The genomic window-size (2.67 Mb) for LD-based scan was set using the average extent of LD decay (average LD_1/10,90_ distance) observed in DDP. This GWAS scan resulted in a set of 254 significant QTL-MTAs from K and QK models covering all traits assessed in the study including QTL-MTAs appearing with multiple gene action models for the same SNP (Supplementary Table S5). Following this, QTL-MTAs appearing with multiple gene action models were filtered to retain only a single QTL-MTA with the highest significance value leading to a non-redundant set of 137 unique QTL-MTAs (Table 5) that forms the basis of GWAS results presented in this study. Graphical representation of QTL-MTAs listed in Table 5 is presented in Supplementary Fig. S6.

**Table 5:** List of significant QTL marker-trait associations (QTL-MTAs) derived using 'K' and 'QK' GWAS models

Trait	Marker	Chrom	Position (bp)	Model	Threshold^a^	− log_10_(*P*) (K)	Effect^b^ (K)	− log_10_(*P*) (QK)	Effect^b^ (QK)
YLD	chr01_84615108	chr01	84615108	general	5.56	6.03	–	–	–
YLD	chr11_4623974	chr11	4623974	general	5.56	5.76	–	6.27	–
MTW	chr01_79074014	chr01	79074014	additive	5.56	7.25	23.15	6.92	23.14
MTW	chr02_21059123	chr02	21059123	additive	5.56	7.92	26.27	7.54	26.27
MTW	chr02_36354621	chr02	36354621	1-dom-alt	5.56	5.82	24.55	5.61	24.46
MTW	chr02_44543919	chr02	44543919	general	5.56	5.58	–	–	–
MTW	chr04_8180136	chr04	8180136	1-dom-alt	5.56	5.98	19.77	–	–
MTW	chr04_8180200	chr04	8180200	additive	5.56	6.64	18.34	6.07	17.89
MTW	chr05_1341916	chr05	1341916	additive	5.56	6.56	25.85	5.96	25.19
MTW	chr05_11242266	chr05	11242266	general	5.56	9.10	–	8.47	–
MTW	chr05_11371658	chr05	11371658	additive	5.56	5.63	20.97	–	–
MTW	chr05_22681150	chr05	22681150	1-dom-alt	5.56	6.00	9.06	5.60	9.01
MTW	chr05_41271797	chr05	41271797	1-dom-alt	5.56	6.67	9.97	6.33	10.01
MTW	chr05_44901864	chr05	44901864	1-dom-alt	5.56	5.67	8.90	–	–
MTW	chr06_54494269	chr06	54494269	additive	5.56	5.63	16.37	–	–
MTW	chr07_3203241	chr07	3203241	general	5.56	6.11	–	5.80	–
MTW	chr08_39791429	chr08	39791429	additive	5.56	7.20	15.07	6.78	14.99
MTW	chr08_45275486	chr08	45275486	additive	5.56	13.26	30.78	12.58	30.99
MTW	chr08_51787959	chr08	51787959	1-dom-alt	5.56	6.41	15.64	5.92	15.43
MTW	chr08_52112043	chr08	52112043	additive	5.56	6.59	10.39	6.15	10.31
MTW	chr08_55923470	chr08	55923470	additive	5.56	6.13	8.84	6.15	9.05
MTW	chr09_2291639	chr09	2291639	additive	5.56	5.69	20.89	–	–
MTW	chr09_6362515	chr09	6362515	1-dom-alt	5.56	7.60	20.61	7.02	20.63
MTW	chr09_6501563	chr09	6501563	general	5.56	8.31	–	7.69	–
MTW	chr09_52666932	chr09	52666932	additive	5.56	6.37	18.27	5.78	17.54
MTW	chr09_67069420	chr09	67069420	1-dom-alt	5.56	5.60	16.61	–	–
MTW	chr11_42310242	chr11	42310242	additive	5.56	5.76	11.10	–	–
MTW	chr11_44871695	chr11	44871695	general	5.56	7.05	–	6.77	–
SHP	chr04_68757669	chr04	68757669	additive	5.56	5.71	0.42	5.83	0.43
REG	chr01_81668820	chr01	81668820	1-dom-ref	5.48	9.85	− 2.36	9.65	− 2.36
REG	chr01_84614836	chr01	84614836	1-dom-ref	5.48	9.85	− 2.36	9.65	− 2.36
REG	chr02_25415640	chr02	25415640	general	5.56	7.29	–	6.67	–
REG	chr09_59884883	chr09	59884883	1-dom-ref	5.48	6.78	− 1.42	6.50	− 1.39
EYE	chr12_1056586	chr12	1056586	1-dom-ref	5.48	5.49	− 1.22	5.56	− 1.23
PUR	chr01_1356581	chr01	1356581	additive	5.56	8.59	− 0.40	7.41	− 0.39
PUR	chr01_1549287	chr01	1549287	1-dom-ref	5.47	7.4	− 0.57	6.69	− 0.55
PUR	chr01_64503675	chr01	64503675	additive	5.56	6.07	− 0.61	5.73	− 0.60
PUR	chr01_65770664	chr01	65770664	1-dom-ref	5.47	6.67	− 0.91	6.59	− 0.91
PUR	chr01_67621461	chr01	67621461	general	5.56	–	–	6.03	NA
PUR	chr01_67671077	chr01	67671077	general	5.56	6.07	NA	–	–
PUR	chr01_71346878	chr01	71346878	1-dom-ref	5.47	6.67	− 0.91	6.59	− 0.91
PUR	chr01_73939403	chr01	73939403	additive	5.56	10.6	− 0.67	10.19	− 0.66
PUR	chr01_78473624	chr01	78473624	1-dom-ref	5.47	6.67	− 0.91	6.59	− 0.91
PUR	chr01_79065553	chr01	79065553	general	5.56	6.12	NA	6.05	NA
PUR	chr02_8566446	chr02	8566446	additive	5.56	7.89	− 0.38	7.84	− 0.38
PUR	chr02_13328431	chr02	13328431	1-dom-ref	5.47	6.89	− 0.93	6.82	− 0.93
PUR	chr02_24220917	chr02	24220917	1-dom-alt	5.56	6.89	0.93	6.82	0.93
PUR	chr02_29489294	chr02	29489294	additive	5.56	8.99	− 0.71	8.92	− 0.71
PUR	chr02_29728440	chr02	29728440	1-dom-ref	5.47	6.92	− 0.77	6.35	− 0.75
PUR	chr02_34625572	chr02	34625572	additive	5.56	5.99	− 0.35	5.89	− 0.35
PUR	chr02_43738555	chr02	43738555	1-dom-ref	5.47	5.67	− 0.96	5.82	− 0.97
PUR	chr02_44077066	chr02	44077066	general	5.56	–	–	5.61	NA
PUR	chr03_251823	chr03	251823	1-dom-ref	5.47	6.99	− 0.93	6.88	− 0.93
PUR	chr03_2108734	chr03	2108734	additive	5.56	7.89	− 0.45	7.59	− 0.44
PUR	chr03_9582227	chr03	9582227	additive	5.56	8.82	− 0.58	8.48	− 0.57
PUR	chr03_52670407	chr03	52670407	general	5.56	7.13	NA	7.04	NA
PUR	chr03_57275947	chr03	57275947	1-dom-ref	5.47	7.01	− 0.80	6.75	− 0.78
PUR	chr03_60220838	chr03	60220838	1-dom-ref	5.47	5.49	− 0.95	5.72	− 0.97
PUR	chr04_944598	chr04	944598	additive	5.56	6.15	− 0.53	6.12	− 0.53
PUR	chr04_59384781	chr04	59384781	1-dom-ref	5.47	6.95	− 0.94	6.79	− 0.93
PUR	chr04_59672854	chr04	59672854	additive	5.56	8.05	− 0.42	6.98	− 0.40
PUR	chr04_62462980	chr04	62462980	1-dom-alt	5.56	6.36	− 0.33	–	–
PUR	chr04_62463076	chr04	62463076	general	5.56	9.65	NA	8.73	NA
PUR	chr05_49212950	chr05	49212950	1-dom-ref	5.47	10.02	− 0.87	9.65	− 0.85
PUR	chr05_51757970	chr05	51757970	1-dom-alt	5.56	7.18	− 0.62	6.88	− 0.61
PUR	chr05_54208577	chr05	54208577	1-dom-ref	5.47	5.88	− 0.72	5.61	− 0.71
PUR	chr06_50515507	chr06	50515507	1-dom-ref	5.47	5.77	− 0.97	5.88	− 0.98
PUR	chr06_52118507	chr06	52118507	additive	5.56	6.12	− 0.59	5.89	− 0.58
PUR	chr06_54208623	chr06	54208623	1-dom-ref	5.47	6.7	− 0.92	6.55	− 0.91
PUR	chr06_57857606	chr06	57857606	general	5.56	7.76	NA	7.71	NA
PUR	chr06_57864175	chr06	57864175	1-dom-ref	5.47	8.38	− 0.46	8.26	− 0.46
PUR	chr06_58253772	chr06	58253772	1-dom-alt	5.56	6.28	− 0.60	6.14	− 0.59
PUR	chr06_58983943	chr06	58983943	additive	5.56	7.35	− 0.36	6.36	− 0.34
PUR	chr07_1780641	chr07	1780641	1-dom-ref	5.47	6.86	− 0.93	6.67	− 0.93
PUR	chr07_6186211	chr07	6186211	1-dom-ref	5.47	6.86	− 0.93	6.67	− 0.93
PUR	chr07_47224659	chr07	47224659	1-dom-ref	5.47	6.86	− 0.93	6.67	− 0.93
PUR	chr07_48818519	chr07	48818519	additive	5.56	8.07	− 0.55	7.79	− 0.54
PUR	chr08_8534417	chr08	8534417	additive	5.56	7.88	− 0.43	7.74	− 0.43
PUR	chr08_41058717	chr08	41058717	1-dom-ref	5.47	7.79	− 0.84	7.68	− 0.84
PUR	chr08_54561995	chr08	54561995	1-dom-ref	5.47	–	–	6.85	− 0.93
PUR	chr08_57420203	chr08	57420203	general	5.56	15.42	NA	14.13	NA
PUR	chr09_55346500	chr09	55346500	1-dom-ref	5.47	6.99	− 0.93	6.98	− 0.94
PUR	chr09_59448889	chr09	59448889	general	5.56	6.71	NA	6.56	NA
PUR	chr09_67278095	chr09	67278095	1-dom-ref	5.47	6.99	− 0.93	6.98	− 0.94
PUR	chr10_54013601	chr10	54013601	general	5.56	6.3	NA	6.02	NA
PUR	chr10_54942916	chr10	54942916	1-dom-ref	5.47	5.56	− 0.54	–	–
PUR	chr11_1026561	chr11	1026561	additive	5.56	6.85	− 0.43	6.85	− 0.43
PUR	chr11_9773425	chr11	9773425	additive	5.56	6.83	− 0.48	6.58	− 0.48
PUR	chr11_46437440	chr11	46437440	general	5.56	5.96	NA	5.96	NA
PUR	chr12_568712	chr12	568712	additive	5.56	6.08	− 0.50	5.9	− 0.50
PUR	chr12_9881625	chr12	9881625	additive	5.56	5.68	− 0.49	–	–
PUR	chr12_53842088	chr12	53842088	1-dom-ref	5.47	6.68	− 0.80	6.15	− 0.77
PUR	chr12_55039992	chr12	55039992	general	5.56	–	–	5.71	NA
PUR	chr12_58144275	chr12	58144275	additive	5.56	8.06	− 0.52	7.9	− 0.51
PUR	chr12_59250968	chr12	59250968	1-dom-ref	5.47	5.72	− 0.45	5.63	− 0.45
TFC	chr03_42577254	chr03	42577254	1-dom-alt	5.56	30.77	1.56	28.89	1.56
TFC	chr03_44063968	chr03	44063968	1-dom-ref	5.48	51.36	− 1.90	48.16	− 1.96
TFC	chr03_45528157	chr03	45528157	1-dom-alt	5.56	43.28	1.81	40.85	1.86
TFC	chr03_45689639	chr03	45689639	additive	5.56	40.54	− 1.50	36.95	− 1.56
TFC	chr03_48721820	chr03	48721820	general	5.56	53.34	–	50.33	–
TSC	chr01_51539256	chr01	51539256	general	5.56	–	–	5.65	–
TSC	chr01_54501268	chr01	54501268	1-dom-ref	5.48	7.83	− 12.34	7.09	− 12.00
TSC	chr01_64394891	chr01	64394891	1-dom-ref	5.48	6.37	12.46	6.28	11.63
TSC	chr01_64608498	chr01	64608498	general	5.56	5.84	–	6.21	–
TSC	chr01_67585923	chr01	67585923	additive	5.56	7.63	2.49	–	–
TSC	chr01_69251218	chr01	69251218	1-dom-ref	5.48	7.83	− 12.34	7.09	− 12.00
TSC	chr01_72387451	chr01	72387451	additive	5.56	6.81	2.54	–	–
TSC	chr01_76492554	chr01	76492554	additive	5.56	7.45	1.98	–	–
TSC	chr01_81279046	chr01	81279046	additive	5.56	7.60	2.54	–	–
TSC	chr02_42067857	chr02	42067857	1-dom-ref	5.48	7.99	− 12.24	7.22	− 12.03
TSC	chr03_33499819	chr03	33499819	1-dom-ref	5.48	6.08	12.20	6.27	11.64
TSC	chr03_42235380	chr03	42235380	additive	5.56	5.64	1.67	–	–
TSC	chr04_13522405	chr04	13522405	additive	5.56	5.68	4.22	–	–
TSC	chr04_60627927	chr04	60627927	1-dom-ref	5.48	6.20	12.22	6.24	11.60
TSC	chr04_62462743	chr04	62462743	general	5.56	5.72	–	5.89	–
TSC	chr06_36626401	chr06	36626401	general	5.56	7.98	–	6.58	–
TSC	chr06_46211337	chr06	46211337	1-dom-ref	5.48	7.87	− 12.06	7.18	− 11.87
TSC	chr06_54454385	chr06	54454385	1-dom-ref	5.48	5.55	− 6.11	–	–
TSC	chr06_58169691	chr06	58169691	1-dom-ref	5.48	7.87	− 12.06	7.18	− 11.87
TSC	chr07_1383345	chr07	1383345	1-dom-ref	5.48	7.64	− 12.06	7.22	− 11.96
TSC	chr07_50704119	chr07	50704119	1-dom-ref	5.48	6.04	12.03	6.23	11.54
TSC	chr07_52738876	chr07	52738876	general	5.56	–	–	5.77	–
TSC	chr08_6944167	chr08	6944167	1-dom-ref	5.48	6.62	12.38	6.36	11.56
TSC	chr08_26608811	chr08	26608811	1-dom-ref	5.48	6.62	12.38	6.36	11.56
TSC	chr08_34574918	chr08	34574918	1-dom-ref	5.48	6.62	12.38	6.36	11.56
TSC	chr08_45486119	chr08	45486119	1-dom-ref	5.48	10.17	− 10.62	10.25	− 10.50
TSC	chr08_59102493	chr08	59102493	1-dom-ref	5.48	6.62	12.38	6.36	11.56
TSC	chr10_58557485	chr10	58557485	general	5.56	8.05	–	6.97	–
TSC	chr12_567852	chr12	567852	1-dom-ref	5.48	8.08	− 12.19	7.38	− 12.00
TST	chr10_11547797	chr10	11547797	general	5.48	–	–	5.57	–
TST	chr10_29388424	chr10	29388424	general	5.48	–	–	5.57	–
POL	chr02_7655142	chr02	7655142	1-dom-alt	5.56	6.02	− 41.55	–	–
POL	chr04_61024485	chr04	61024485	general	5.56	–	–	5.63	–
POL	chr05_23152674	chr05	23152674	1-dom-ref	5.48	5.63	− 37.83	–	–
POL	chr06_35636880	chr06	35636880	additive	5.56	6.41	− 28.74	5.94	− 28.12
POL	chr09_5184271	chr09	5184271	additive	5.56	6.32	− 37.19	5.79	− 35.77
POL	chr12_55741943	chr12	55741943	1-dom-ref	5.48	6.36	− 33.90	6.33	− 33.89

For each trait, only a single MTA with the highest significance value per LD-based genomic interval was retained for SNPs appearing with multiple gene action models

YLD, Yield; MTW, Mean tuber weight; SHP, Tuber shape; REG, Regularity of tuber shape; EYE, Tuber eye depth; PUR, purple tuber skin colour; TFC, Tuber flesh colour; TSC, Tuber starch content; TST, Boiled tuber taste; POL, Pollen fertility

^a^Genome-wide* P*-value (− log_10_(*P*)) detection threshold for statistical significance

^b^Marker effect (not available for the general model because there are multiple effects)

Significant QTL-MTAs were detected for all ten traits tested in DDP (Table 5 and Supplementary Table S5; Fig. 7 and Supplementary Fig. S6). A marker from chromosome 1 (84.62 Mb) and another one from chromosome 11 (4.62 Mb) were associated with YLD according to GWAS model K. The association on chromosome 11 was also significant for YLD according to GWAS model QK. Significant associations for MTW were found on nine chromosomes: 1, 2, 3, 4, 6, 7, 8, 9 and 11. The strongest association with this trait was identified on chromosome 8 at 45.28 Mb, with − log_10_(*P*) values reaching 13.26 and 12.58 according to GWAS models K and QK, respectively. A single marker located on chromosome 4 at 68.76 Mb was significantly associated with SHP according to both GWAS models. For REG, significant QTL-MTAs were identified on chromosomes 1, 2 and 9 with the ones on chromosome 1 being strongest. Two markers located at 81.67 and 84.61 Mb on chromosome 1 showed the association with REG at the − log_10_(*P*) level of 9.85 or 9.65 depending on GWAS model. For EYE, similarly as for SHP, only one marker was significant. It was located at 1.06 Mb on chromosome 12. Significant QTL-MTAs for PUR were detected on all chromosomes. The strongest of them was with a marker on chromosome 8 (57.42 Mb), significant at the -log_10_(*P*) levels 15.42 and 14.13, according to K and QK GWAS models, respectively. The most significant association among all tested traits in our study was detected between a marker located on chromosome 3 at 48.72 Mb and TFC. The − log_10_(*P*) level reached 53.34 (K model) or 50.33 (QK model) for this QTL-MTA. Markers significantly associated with TFC were distributed on chromosome 3 between 42.58 and 48.72 Mb. TSC was affected by a number of loci located on nine chromosomes: 1, 2, 3, 4, 6, 7, 8, 10 and 12. The strongest association with TSC was identified on chromosome 8 at 45.49 Mb, with − log_10_(*P*) values reaching 10.17 and 10.25 according to GWAS models K and QK, respectively. Two QTL-MTAs located on chromosome 10 at 11.55 and 29.39 Mb were significantly associated with TST according to the QK model, both with − log_10_(*P*) 5.57. For POL, significant QTL-MTAs were detected on six chromosomes: 2, 4, 5, 6, 9 and 12. The QTL-MTAs from chromosome 6 were the strongest for POL with − log_10_(*P*) 6.41 and 5.94 according to K and QK GWAS models, respectively (Table 5, Fig. 7).

## Discussion

### QTL corroborations and new findings

DDP used in this study is a collection of diploid lines with various wild germplasm introgressions in an *S. tuberosum* dihaploid background. The lines present in DDP or closely related ones, have been used previously in linkage studies on inheritance of traits such as tuber morphology (Śliwka et al. 2008; Hara-Skrzypiec et al. 2018a), tuber starch content (Śliwka et al. 2016; Hara-Skrzypiec et al. 2018b; Sołtys-Kalina et al. 2020; Lebecka et al. 2021), chip colour and resistances to various diseases. In the current GWAS, we found some QTL located in genomic regions described in the earlier studies using plant material related to, or included in, DDP. We also detected some QTL likely caused by widespread *S. tuberosum-*derived alleles described by other authors. The introgressions of wild *Solanum* species in DDP in our study resulted in finding some QTL in new locations.

The QTL for YLD have not been mapped in the studies done in related material but of the two QTL identified in this study on chromosomes 1 and 11, the one on chromosome 11 identified by the marker located at 4.62 Mb (Table 5) seems to be the same as the QTL identified by a marker at 5.40 Mb described by Garzón-Martínez et al. (2024) in a GWAS using 568 *S. tuberosum* group Andigenum accessions. Accessions of Andigenum group were in the pedigree of DDP as donors of the virus and nematode resistances. Garzón-Martínez et al. (2024) also discovered QTL for yield in several regions of chromosome 1, but none of them is close to position of the QTL identified in our work at 84.62 Mb. A QTL for yield has been described in a similar region at 78.8 Mb by Sharma et al. (2024) in a GWAS of tetraploid potato panel. Our QTL may also overlap with the one described by Bradshaw et al. (2008) at the bottom of chromosome 1 (118 cM), but due to different genotyping systems precise comparison of location is not possible.

We detected QTL for MTW on chromosomes 1, 2, 4, 5, 6, 7, 8, 9 and 11 and the ones on chromosomes 1, 4, 5 and 6 are likely similar to those described by Hara-Skrzypiec et al. (2018a). The QTL on chromosome 2 with significant QTL-MTAs at 36.35 and 44.54 Mb spans over the region of 40.21 Mb indicated as significant for the average tuber weight by Sharma et al. (2024). The tuber weight or size has been studied in several other works (e.g. Braun et al. 2017; Manrique-Carpintero et al. 2015), but the QTL for MTW on chromosomes 8, 9 and 11 have not been described earlier.

For SHP a single QTL was detected on chromosome 4 but in a position most likely different from the position of QTL for SHP described by Hara-Skrzypiec et al. (2018a). However, a QTL for SHP has been described by Zia et al. (2020) in a GWAS performed in a panel of 237 tetraploid table potato cultivars, as located on chromosome 4 at 65.07 Mb in a position similar to the position of the QTL identified in this study at 68.76 Mb. The only QTL for EYE detected in our study on chromosome 12 at 1.06 Mb was not described earlier in the related material, but it is in the similar position as the QTL described by Sharma et al. (2024) at 1.45 Mb. Surprisingly, the major loci for SHP and EYE described on chromosome 10 (van Eck et al. 1994; van Eck 2007; Lindqvist-Kreuze et al. 2015; Sharma et al. 2024) were not significant in our study. Nevertheless, candidate QTL peaks for tuber shape and eye depth (Fig. 7) were observed on chromosome 10 in the current study. Interestingly, the QTL-MTAs with highest significance in these candidate peaks for SHP (chr10_47842697 and chr10_50731367) were in the known locus on chromosome 10 while those for the linked EYE trait (chr10_60478479, chr10_58557485 and chr10_59064804) were located around 8–10 Mb distance apart (Supplementary Table S6). Moreover, two of the tuber shape (chr10_50383861 and chr10_49714737) and one eye depth (chr10_50383861) chromosome 10 MTAs reported by Sharma et al. (2024) were present in DDP but not positioned in the top part of the candidate peaks observed for these two traits (Supplementary Table S6). These three QTL-MTAs with MAFs of 0.39, 0.36 and 0.39, respectively, along with most other QTL-MTAs observed in the chromosome 10 tuber shape and eye depth QTL region as reported by Sharma et al. (2024), displayed marker properties (Supplementary Fig. S2) in the dynamic range suitable for performing GWAS leaving the results intriguing. The reduced statistical power to detect significant QTL for SHP and EYE in the described chromosome 10 genomic regions reported in cultivated gene pool could be due to the potential introgression of other independent association signals and loci from wild potato relatives exerting genetic effects on tuber shape and eye depth. The dissociation of chromosome 10 candidate peak MTAs for SHP and EYE as described above further suggests the potential perturbance in LD for this region due to broadening of the tuberosum gene pool. Notably, in linkage studies performed on material related to DDP, QTL for these two traits have been mapped with chromosome 10 QTL either strongly affecting EYE and slightly SHP (Śliwka et al. 2008) or strongly affecting SHP only (Hara-Skrzypiec et al. 2018a) suggesting these dominant alleles were segregating in the described linkage studies. This further suggests that these dominant alleles affecting SHP and EYE are present in DDP but perhaps in lower frequencies giving decreased power for GWAS.

GWAS for REG identified significant QTL-MTAs on chromosomes 1, 2 and 9, however, none of these was corroborating the positions of QTL detected in the previous linkage studies (Śliwka et al. 2008; Hara-Skrzypiec et al. 2018a) performed in related material. Moreover, unlike in previous studies, the QTL detected in GWAS for SHP, REG and EYE did not overlap with each other.

We detected QTL for PUR on all potato chromosomes including the locations corresponding to three well-described loci affecting anthocyanin pigmentation in potato. Even though locus *R* (*D*) on chromosome 2 is associated with red anthocyanin pigmentation in potato (van Eck et al. 1993, 1994), it may also affect the purple pigmentation of tuber skin analysed in our study. Interestingly, the genomic positions (34.6 Mb and 43.7 Mb) of the two chromosome 2 significant QTA-MTAs for PUR observed here spans the location (38.13 Mb on chromosome 2; Pham et al. 2020; Potato Genome Sequencing Consortium 2011) of the *dihydroflavonol 4-reductase* gene Soltu.DM.02G024900 known to be associated with locus *R* (*D*) (De Jong et al. 2003; Zhang et al. 2009) described above. Anthocyanin pigmentation of tuber skin in potato has also been associated with locus *D* (also known as *I* in diploid potato or *PSC*) on chromosome 10 (van Eck et al. 1994). The* D* locus encodes an R2R3 MYB transcription factor similar to *Petunia an2* which in potato is named as *Stan2* (De Jong et al. 2004; Jung et al. 2009) and located (52.60 Mb on chromosome 10; Soltu.DM.10G020850; Pham et al. 2020; Potato Genome Sequencing Consortium 2011) in close vicinity to the two chromosome 10 significant QTL-MTAs (at 54.01 Mb and 54.94 Mb) for PUR detected in our study implicating that *Stan2* may underlie this QTL. The third locus important for blue anthocyanin pigmentation in potato is the *P* locus on chromosome 11 (van Eck et al. 1994) encoding flavonoid 3′,5′-hydroxylase (Jung et al. 2005). The best match to *flavonoid 3′,5′-hydroxylase* in the reference potato genome is Soltu.DM.11G020990 (40.98 Mb on chromosome 11; Pham et al. 2020; Potato Genome Sequencing Consortium 2011) which is located within ~ 6 Mb of a QTL for PUR detected in our study at 46.44 Mb. Pandey et al. (2022) have described QTL for purple tuber skin on chromosomes 1, 2, 3, 4, 10 and 11 using an association panel of 214 tetraploid potato clones and cultivars. All of them overlap with the QTL for PUR identified in our study. Parra-Galindo et al. (2019, 2021) have described QTL for contents of different anthocyanidins in tubers of diploid landrace accessions from the *S. tuberosum* Group Phureja on chromosomes 1, 2, 6, 9, 10, 11 and 12. The QTL for PUR described in our study are in similar positions as the QTL on chromosomes 2, 9, 10 and 11 reported in these two studies but precise comparisons of the QTL locations are hampered by the use of different versions of the reference genome. Caraza-Harter (2020) has mapped QTL for red pigmentation of potato tuber periderm (skin hue, skin lightness and skinned area hue) in a tetraploid potato panel on chromosomes 1, 2, 5, 9, 10 and 11. These QTL also overlap with the QTL for PUR observed here, with the exception of regions on chromosomes 5 (3.87 Mb) and 9 (8.31 Mb) that were not significantly associated with tuber skin pigmentation in our study. These results indicate that the genetic factors affecting purple or red tuber skin colour are to a large extent common. Besides several previously described QTL, the current study detected new QTL for PUR on chromosomes 6, 7 and 8, with the QTL on chromosome 8 being the most significant among all PUR QTLs observed here.

An example of QTL detected both in *S. tuberosum* germplasm and in previous studies conducted using DDP-related material, is the QTL for TFC on chromosome 3 detected in DDP between 42.58 and 48.72 Mb and overlapping with the position of a *beta-carotene hydroxylase* gene Soltu.DM.03G018410 at 42.90 Mb of the chromosome 3 in the reference genome DMv6.1 (Pham et al. 2020; Potato Genome Sequencing Consortium 2011). The *beta-carotene hydroxylase* gene has been indicated as the gene underlying the *Y* (*Yellow*) locus determining yellow flesh colour in potato (Kloosterman et al. 2010). The *Y* locus has been described in early linkage mapping studies (Bonierbale et al. 1988; Brown et al. 1993, 2006). QTL for TFC has been described in a location corresponding to the *Y* locus in the recent GWAS study using European tetraploid potato germplasm, as one of the seven detected QTL for this trait (Sharma et al. 2024). The same QTL was detected in linkage studies performed on diploid plant material related to DDP (Śliwka et al. 2008; Hara-Skrzypiec et al. 2018a). However, in both linkage studies the QTL on chromosome 3 was not the only one affecting TFC, in contrast to the current GWAS study. Śliwka et al. (2008) described QTL for TFC on chromosomes 2, 4, 11 and 12, and the QTL on chromosome 3 with only a minor effect on TFC, which led to the hypothesis that both parents of this mapping population did not have the dominant *Y* allele. In a different biparental population, Hara-Skrzypiec et al. (2018a) detected a QTL in the region corresponding to *beta-carotene hydroxylase* location that explained 76.8% of variance in TFC, and an additional QTL on chromosome 2. In the GWAS study of DDP the strong effect of the QTL on chromosome 3 for the TFC potentially covered the effects of other loci on this trait.

There are numerous studies mapping TSC in potato, including four performed using plant material closely related to DDP, all confirming polygenic inheritance of the trait. In this study we found QTL for TSC on chromosomes 1, 2, 3, 4, 6, 7, 8, 10 and 12, all in positions overlapping with the QTL described in from one to three of the previous studies (Śliwka et al. 2016; Hara-Skrzypiec et al. 2018b; Sołtys-Kalina et al. 2020; Lebecka et al. 2021).

### GWAS findings for previously unmapped traits

Taste (flavour) of boiled potato tubers is a trait important for consumers and breeders, but complex and difficult to score. Differences in taste tend to be confounded with differences in texture which is another complex trait with several parameters: disintegration, consistency, mealiness, dryness and structure. Potato cultivars can be categorized into cooking types A–D according to their texture parameters (Domański 2001b). The scoring of TST in this study aimed to assess flavour on the scale from very bad to very good, independent of cooking type or texture. Using the DDP, we identified two regions of chromosome 10 at 11.55 Mb and 29.39 Mb as affecting the TST. Which genes are underlying these QTL can be a difficult question to answer as numerous soluble and volatile compounds may influence potato flavour (Taylor et al. 2007). Glutamic acid, other amino acids, guanosine-5’-monophosphate and other 5’-ribonucleotides have been indicated as main determinants of boiled potato flavour (Solms 1971). Glutamate and guanosine-5’-monophosphate have been demonstrated as the compounds responsible for the umami taste and higher flavour scores of the mature, cooked *S. tuberosum* group Phureja potatoes (Morris et al. 2007). The content of glycoalkaloids, low in table potato cultivars, have had no effect on taste of boiled or steamed tubers, but bitter-tasting free amino acids and tyrosine derivative, homogentisic acid, have been indicated as causal for egumi taste described as “unpleasantly harsh, somewhat bitter, and astringent taste” in Japan (Sato et al. 2019). Drapal et al. (2023) have listed 39 metabolites changing in tubers during potato storage and associated with sweetness (e.g. glucose, fructose, glycine and serine), sourness (oxalic, ascorbic and citric acids) and umami (aspartic acid), as well as affecting the overall ratings of potato flavour. Our results provide a starting point for studying the genes affecting the differences in boiled tuber taste and contents of metabolites important for this trait.

Lactofuchsin staining of potato pollen has been used as a pollen fertility estimate (POL) at IHAR-PIB for a long time and proved to be useful in diploid breeding practice. We mapped six QTL for POL on chromosomes 2, 4, 5, 6, 9 and 12. The fertility of diploid potatoes gains significance due to the development of diploid breeding programs deploying self-compatibility and hybrid breeding strategy. Fertility is one of the traits severely affected by inbreeding depression in potato and some recent studies aimed at describing its genetics. Pollen viability was evaluated and segregated in three progenies developed by Zhang et al. (2019) by selfing, but they did not identify major loci for this trait. Using binary classification (poor or good pollen producer) Clot et al. (2024a) mapped pollen shed to seven QTL located on chromosomes 1, 2, 5, 9, 10 and 11, with the QTL *PSE2* on chromosome 2 indicated as the most important. Position of the QTL on chromosome 9 at 1.95 Mb (Clot et al. 2024a) is similar to the position of the QTL detected for POL in our study at 5.56 Mb of the chromosome 9, while the other QTL do not overlap. A different pollen staining method, but also containing acid fuchsin (Peterson et al. 2010), has been used to phenotype crossover shortage and to map it to the short arm of chromosome 8 (Clot et al. 2024b). *StMSH4* has been shown to be encoded in this locus and its mutation as leading to either unreduced pollen or sterility. The gene is a potato ortholog of one of the genes from the class I crossover pathway (ZMM pathway) responsible for 75–85% of crossovers in *Arabidopsis thaliana.* In the potato reference genome, ten orthologs of the *A. thaliana* ZMM genes have been identified (Clot 2023). Among them, *StZYP1* (*Soltu.DM.04G029680*) is located at 61.02 Mb on chromosome 4, overlapping very closely with the position of the chromosome 4 QTL-MTA for POL detected in our study (Table 5), which makes it a plausible candidate gene for pollen fertility.

In recent years, genome-wide association studies have emerged as a powerful tool for the genetic analysis of complex traits in many organisms and offer several advantages over the use of other population types (e.g. biparental populations) not least the ability to analyse large number of phenotyped traits simultaneously. In potato, most GWAS studies have been conducted using the panel comprising cultivated tetraploid potato genotypes. Two recent studies have been performed using diploid material of the Phureja group (Díaz et al. 2021) or ethyl methanosulphonate-mutagenized clones (Fofana et al. 2024), and there is one article describing a mixed GWAS panel of 320 tetraploid and 84 diploid potato accessions of mostly Colombian origins (Berdugo-Cely et al. 2023). However, the current study deploys a unique diploid diversity panel with a widened cultivated potato gene pool obtained as a result of introgressing valuable traits from wild potato relatives and the cultivated Andean potato species to the *S. tuberosum* background. Considering the increased interest in diploid hybrid potato breeding, the results presented here hold greater relevance and provide novel targets for potato breeding and research at the diploid level.

## Supplementary Information

Below is the link to the electronic supplementary material.

Supplementary Fig. S1 Overall distribution of (a) minor allele frequency (MAF), (b) maximum genotype frequency, and (c) polymorphism information content (PIC) for 39,756 GBS SNPs used in the study (PNG 458 KB)

Supplementary Fig. S2 Chromosome-wise distribution of (a) minor allele frequency (MAF), (b) maximum genotype frequency, and (c) polymorphism information content (PIC) for 39,756 GBS SNPs used in the study. SNPs located in the chromosome 10 tuber shape and eye depth QTL region as reported by Sharma et al. (2024) are highlighted in red (PNG 15765 KB)

Supplementary Fig. S3 Screeplot from the Principal Component Analysis (PCA) displaying the number of principal components versus their corresponding eigenvalues. PCA performed using 39,756 GBS SNPs (PNG 376 KB)

Supplementary Fig. S4 Bayesian information criterion (BIC) statistical measure of goodness of fit curve for detecting optimal number of clusters (k-means) or subpopulations (Q) in DDP obtained using 39,756 GBS SNPs (PNG 301 KB)

Supplementary Fig. S5 Q-Q plots comparing the inflation of p-values for the four principal GWAS models deployed for each genetic (gene action) model for all 10 traits (YLD, Yield; MTW, Mean tuber weight; SHP, Tuber shape; REG, Regularity of tuber shape; EYE, Tuber eye depth; PUR, Purple tuber skin colour; TFC, Tuber flesh colour; TSC, Tuber starch content; TST, Boiled tuber taste; POL, Pollen fertility). Red circles: Naïve model; Green squares: K model; Blue diamonds: Q model; and Black triangles: QK model. Red line indicates p-values under the expected normal distribution (PNG 4762 KB)

Supplementary Fig. S6 Graphical illustration of the non-redundant set of unique QTL-MTAs (a, K models; b, QK models) listed in Table 5 (trait codes: YLD, Yield; MTW, Mean tuber weight; SHP, Tuber shape; REG, Regularity of tuber shape; EYE, Tuber eye depth; PUR, Purple tuber skin colour; TFC, Tuber flesh colour; TSC, Tuber starch content; TST, Boiled tuber taste; POL, Pollen fertility). Black dashed line depicts GWAS main significance threshold set to 1e-5 - log10(p) value while actual significance thresholds obtained separately for each trait MTA using Bonferroni-type multiple testing correction method “M.eff” (genome-wide α = 0.05) are provided in Table 5. The chromosome heatmaps below each plot illustrate SNP density (bin size = 1 Mb) (PNG 2711 KB)

Supplementary Table S1 List of potato diploid diversity panel genotypes employed in the study including their population group (cluster) membership details and main breeding aims (33 KB)

Supplementary Table S2 List of barcodes employed to construct 96-plex GBS libraries (11 KB)

Supplementary Table S3 List of genes overlapping GBS SNPs including impact category count (1917 KB)

Supplementary Table S4 Genomic control inflation factor (λGC) values for each trait and 'genetic model x GWAS model' combination. Trait codes: YLD, Yield; MTW, Mean tuber weight; SHP, Tuber shape; REG, Regularity of tuber shape; EYE, Tuber eye depth; PUR, Purple tuber skin colour; TFC, Tuber flesh colour; TSC, Tuber starch content; TST, Boiled tuber taste; POL, Pollen fertility (16 KB)

Supplementary Table S5 List of significant QTL marker-trait associations (QTL-MTAs) detected in diploid diversity panel (DDP). QTL-MTAs appearing for all gene action models are included. Trait codes: YLD, Yield; MTW, Mean tuber weight; SHP, Tuber shape; REG, Regularity of tuber shape; EYE, Tuber eye depth; PUR, Purple tuber skin colour; TFC, Tuber flesh colour; TSC, Tuber starch content; TST, Boiled tuber taste; POL, Pollen fertility (29 KB)

Supplementary Table S6 List of (a) top QTL-MTAs detected in tuber shape and tuber eye depth candidate GWAS peaks and (b) QTL-MTAs for these two traits common between DDP and Sharma et al. (2024). Cells highlighted in grey contain the most significant -log10(p) values corresponding to the respective gene action models listed under column ‘Ranking Model’ (13 KB)

The legends for the Supplementary files (20 KB)

## Data Availability

The genotype and phenotype information used for this study is available at figshare: 10.6084/m9.figshare.28219718. Plant material is available from National Centre for Plant Genetic Resources: Polish Genebank: https://bankgenow.edu.pl/en/.

## Abbreviations

DDP: Diploid diversity panel
EYE: Tuber eye depth
GBS: Genotyping-by-sequencing
GWAS: Genome-wide association study
LD: Linkage disequilibrium
MAF: Minor allele frequency
MTA: Marker-trait association
MTW: Mean tuber weight
POL: Pollen fertility
PUR: Purple tuber skin colour
QTL: Quantitative trait loci
REG: Regularity of tuber shape
SHP: Tuber shape
SNP: Single-nucleotide polymorphism
TFC: Tuber flesh colour
TSC: Tuber starch content
TST: Boiled tuber taste
YLD: Yield

## References

[CR1] Abdalla MMF (1970) Inbreeding, heterosis, fertility, plasmon differentiation and *Phytophthora* resistance in *Solanum verrucosum* Schlechtd., and some interspecific crosses in *Solanum*. PhD Thesis. Agric. Res. Rep. Wageningen 748 pp 1–213

[CR2] Anonymous (1974) Ocena wartości konsumpcyjnej ziemniaków. Instrukcja. Z Prac Instytutu Ziemniaka Biuletyn Informacyjny 5:1–8

[CR3] Berdugo-Cely JA, Céron-Lasso MDS, Yockteng R (2023) Phenotypic and molecular analyses in diploid and tetraploid genotypes of Solanum tuberosum L. reveal promising genotypes and candidate genes associated with phenolic compounds, ascorbic acid contents, and antioxidant activity. Front Plant Sci 13:1007104. 10.3389/fpls.2022.1007104 36743552 10.3389/fpls.2022.1007104 PMC9889998

[CR4] Bethke PC, Halterman DA, Jansky S (2017) Are we getting better at using wild potato species in light of new tools? Crop Sci 57:1241–1258. 10.2135/cropsci2016.10.0889

[CR5] Bolger AM, Lohse M, Usadel B (2014) Trimmomatic: a flexible trimmer for Illumina sequence data. Bioinformatics 30:2114–2120. 10.1093/bioinformatics/btu170 24695404 10.1093/bioinformatics/btu170 PMC4103590

[CR6] Bonierbale MW, Plaisted RL, Tanksley SD (1988) RFLP maps based on common set of clones reveal modes of chromosomal evolution in potato and tomato. Genetics 120:1095–1103. 10.1093/genetics/120.4.1095 17246486 10.1093/genetics/120.4.1095 PMC1203572

[CR7] Bradshaw JE (2022) Breeding diploid F1 hybrid potatoes for propagation from botanical seed (TPS): comparisons with theory and other crops. Plants 11:1121. 10.3390/plants11091121 35567122 10.3390/plants11091121 PMC9101707

[CR8] Bradshaw JE, Hackett CA, Pande B, Waugh R, Bryan GJ (2008) QTL mapping of yield, agronomic and quality traits in tetraploid potato (*Solanum tuberosum* subsp. *tuberosum*). Theor Appl Genet 116:193–211. 10.1007/s00122-007-0659-1 17938877 10.1007/s00122-007-0659-1

[CR9] Braun SR, Endelman JB, Haynes KG, Jansky SH (2017) Quantitative trait loci for resistance to common scab and cold-induced sweetening in diploid potato. Plant Genome 10:3. 10.3835/plantgenome2016.10.0110 10.3835/plantgenome2016.10.0110 29293805

[CR10] Brown CR, Edwards CG, Yang C-P, Dean BB (1993) Orange flesh trait in potato: inheritance and carotenoid content. J Am Soc Hortic Sci 118:145–150

[CR11] Brown CR, Kim TS, Ganga Z, Haynes K, De Jong D, Jahn M, Paran I, De Jong W (2006) Segregation of total carotenoid in high level potato germplasm and its relationship to beta-carotene hydroxylase polymorphism. Am J Potato Res 83(5):365–372. 10.1007/BF02872013

[CR12] Caraza-Harter MV (2020) Genetics of skin set and color in red potatoes. PhD thesis, University of Wisconsin-Madison, USA. https://digital.library.wisc.edu/1711.dl/HPT7SA5DMDPHL85

[CR13] Chase SS (1963) Analytic breeding in *Solanum tuberosum* L. – a scheme utilizing parthenotes and other diploid stocks. Can J Genet Cytol 5:359–363. 10.1139/g63-049

[CR14] Clot CR, Polzer C, Prodhomme C, Schuit C, Engelen CJM, Hutten RCB, van Eck HJ (2020) The origin and widespread occurrence of *Sli*-based self-compatibility in potato. Theor Appl Genet 133:2713–2728. 10.1007/s00122-020-03627-8 32514711 10.1007/s00122-020-03627-8 PMC7419354

[CR15] Clot CR, Wang X, Koopman J, Navarro AT, Bucher J, Visser RG, Finkers R, van Eck HJ (2024a) High-density linkage map constructed from a skim sequenced diploid potato population reveals transmission distortion and QTLs for tuber yield and pollen shed. Pot Res 67:139–163. 10.1007/s11540-023-09627-7

[CR16] Clot CR, Klein D, Koopman J, Schuit C, Engelen CJM, Hutten RCB, Brouwer M, Visser RGF, Jurani M, van Eck HJ (2024b) Crossover shortage in potato is caused by *StMSH4* mutant alleles and leads to either highly uniform unreduced pollen or sterility. Genetics. 10.1093/genetics/iyad194 37943687 10.1093/genetics/iyad194 PMC10763545

[CR17] Clot C (2023) Natural variation in potato sexual reproduction facilitates breeding. PhD thesis, Wageningen University, Wageningen, the Netherlands

[CR18] De Jong WS, De Jong DM, De Jong H, Kalazich J, Bodis M (2003) An allele of dihydroflavonol 4-reductase associated with the ability to produce red anthocyanin pigments in potato (Solanum tuberosum L.). Theor Appl Genet 107(8):1375–1383. 10.1007/s00122-003-1395-9 12955207 10.1007/s00122-003-1395-9

[CR19] De Jong WS, Eannetta NT, De Jong DM, Bodis M (2004) Candidate gene analysis of anthocyanin pigmentation loci in the Solanaceae. Theor Appl Genet 108(3):423–432. 10.1007/s00122-003-1455-1 14523517 10.1007/s00122-003-1455-1

[CR20] DePristo MA, Banks E, Poplin R, Garimella KV, Maguire JR et al (2011) A framework for variation discovery and genotyping using next-generation DNA sequencing data. Nat Genet 43:491–498. 10.1038/ng.806 21478889 10.1038/ng.806 PMC3083463

[CR21] Díaz P, Sarmiento F, Mathew B, Ballvora A, Mosquera Vásquez T (2021) Genomic regions associated with physiological, biochemical and yield-related responses under water deficit in diploid potato at the tuber initiation stage revealed by GWAS. PLoS ONE 16(11):e0259690. 10.1371/journal.pone.0259690 34748612 10.1371/journal.pone.0259690 PMC8575265

[CR22] Domański L (2001a) Assessment of morphological characters of potato tubers. Monografie i Rozprawy Naukowe IHAR, Radzików, Poland 10:92–95

[CR23] Domański L (2001b) Assessment of cooking quality of potatoes. Monografie i Rozprawy Naukowe IHAR, Radzików, Poland 10:96–100

[CR24] Drapal M, De Boeck B, Lindqvist Kreuze H, Bonierbale M, Fraser PD (2023) Identification of metabolites associated with boiled potato sensory attributes in freshly harvested and stored potatoes. J Food Compos Anal 115:104934. 10.1016/j.jfca.2022.104934

[CR25] FAOSTAT. https://www.fao.org/faostat/en/#home. Accessed on 13 February 2024

[CR26] Fofana B, Soto-Cerda B, Zaidi M, Main D, Fillmore S (2024) Genome-wide genetic architecture for plant maturity and drought tolerance in diploid potatoes. Front Genet 14:1306519. 10.3389/fgene.2023.1306519 38357658 10.3389/fgene.2023.1306519 PMC10864671

[CR27] Garzón-Martínez GA, Azevedo CF, Berdugo-Cely JA, Lasso-Paredes ZL, Coronel-Ortiz B, Ferrão LFV, Enciso-Rodríguez FE (2024) Genetic dissection of yield and quality-related traits in a Colombian Andigenum potato collection, revealed by genome-wide association and genomic prediction analyses. Euphytica 220:79. 10.1007/s10681-024-03337-y

[CR28] Hara-Skrzypiec A, Śliwka J, Jakuczun H, Zimnoch-Guzowska E (2018a) QTL for tuber morphology traits in diploid potato. J Appl Genet 59(2):123–132. 10.1007/s13353-018-0433-x 29492845 10.1007/s13353-018-0433-x PMC5895667

[CR29] Hara-Skrzypiec A, Śliwka J, Jakuczun H, Zimnoch-Guzowska E (2018b) Quantitative trait loci for tuber blackspot bruise and enzymatic discoloration susceptibility in diploid potato. Mol Genet Genomics 293(2):331–342. 10.1007/s00438-017-1387-0 29080143 10.1007/s00438-017-1387-0 PMC5854731

[CR30] Hosaka K, Hanneman RE (1998) Genetics of self-compatibility in a self-incompatible wild diploid potato species *Solanum chacoense*. 1. Detection of an *S locus inhibitor* (*Sli*) gene. Euphytica 99:191–197. 10.1023/A:1018353613431

[CR31] Huamán Z, Williams JT, Salhuana W, Vincent L (1977) Descriptors for the cultivated potato and for the maintenance and distribution of germplasm collections. International Board for Plant Genetic Resources, Rome, Italy

[CR32] Janssen AWB, Hermsen JGT (1976) Estimating fertility in *Solanum* species and haploids. Euphytica 25:577–586. 10.1007/BF00041595

[CR33] Jung CS, Griffiths HM, De Jong DM, Cheng S, Bodis M, De Jong WS (2005) The potato *P* locus codes for flavonoid 3′, 5′-hydroxylase. Theor Appl Genet 110:269–275. 10.1007/s00122-004-1829-z 15565378 10.1007/s00122-004-1829-z

[CR34] Jung CS, Griffiths HM, De Jong DM, Cheng S, Bodis M, Kim TS, De Jong WS (2009) The potato developer (*D*) locus encodes an R2R3 MYB transcription factor that regulates expression of multiple anthocyanins structural genes in tuber skin. Theor Appl Genet 120:45–57. 10.1007/s00122-009-1158-3 19779693 10.1007/s00122-009-1158-3 PMC2778721

[CR35] Kloosterman B, Oortwijn M, Willigen JU, America T, de Vos R, Visser RGF, Bachem CWB (2010) From QTL to candidate gene: genetical genomics of simple and complex traits in potato using a pooling strategy. BMC Genomics 11:158. 10.1186/1471-2164-11-158 20210995 10.1186/1471-2164-11-158 PMC2843620

[CR36] Koenker R (2017) quantreg: Quantile Regression. R package version 5.34 edn https://CRAN.R-project.org/package=quantreg

[CR37] Langmead B, Salzberg SL (2012) Fast gapped-read alignment with Bowtie 2. Nat Methods 9:357–359. 10.1038/nmeth.1923 22388286 10.1038/nmeth.1923 PMC3322381

[CR38] Lebecka R, Śliwka J, Grupa-Urbańska A, Szajko K, Marczewski W (2021) QTLs for potato tuber resistance to *Dickeya solani* are located on chromosomes II and IV. Plant Pathol 70(7):1745–1756. 10.1111/ppa.13407

[CR39] Lindqvist-Kreuze H, Khan A, Salas E, Meiyalaghan S, Thompson S, Gomez R, Bonierbale M (2015) Tuber shape and eye depth variation in a diploid family of Andean potatoes. BMC Genet 16:57. 10.1186/s12863-015-0213-0 26024857 10.1186/s12863-015-0213-0 PMC4448561

[CR40] Lunden PA (1956) Underldokerd over forholder mellom popetens spesifikka vekt og deres torvstoff og Stivelsesinhold Forhl. Forsok Landbruket 7:81–107

[CR41] Manrique-Carpintero NC, Coombs JJ, Cui YH, Veilleux RE, Buell CR, Douches D (2015) Genetic map and QTL analysis of agronomic traits in a diploid potato population using single nucleotide polymorphism markers. Crop Sci 55:2566–2579. 10.2135/cropsci2014.10.0745

[CR42] McKenna A, Hanna M, Banks E, Sivachenko A, Cibulskis K et al (2010) The genome analysis toolkit: a mapreduce framework for analyzing next-generation DNA sequencing data. Genome Res 20:1297–1303. 10.1101/gr.107524.110 20644199 10.1101/gr.107524.110 PMC2928508

[CR43] Melo ATO, Bartaula R, Hale I (2016) GBS-SNP-CROP: a reference-optional pipeline for SNP discovery and plant germplasm characterization using variable length, paired-end genotyping-by-sequencing data. BMC Bioinformatics 17:29. 10.1186/s12859-016-0879-y 26754002 10.1186/s12859-016-0879-y PMC4709900

[CR44] Morris WL, Ross HA, Ducreux LJM, Bradshaw JE, Bryan GJ, Taylor MA (2007) Umami compounds are a determinant of the flavor of potato (*Solanum tuberosum* L.). J Agric Food Chem 55:9627–9633. 10.1021/jf0717900 17944535 10.1021/jf0717900

[CR45] Pandey J, Scheuring DC, Koym JW, Vales MI (2022) Genomic regions associated with tuber traits in tetraploid potatoes and identification of superior clones for breeding purposes. Front Plant Sci 13:952263. 10.3389/fpls.2022.952263 35937326 10.3389/fpls.2022.952263 PMC9354404

[CR46] Parra-Galindo MA, Piñeros-Niño C, Soto-Sedano JC, Mosquera- Vásquez T (2019) Chromosomes I and X harbor consistent genetic factors associated with the anthocyanin variation in potato. Agronomy 9:11–13. 10.3390/agronomy9070366

[CR47] Parra-Galindo MA, Soto-Sedano JC, Mosquera-Vásquez T, Roda F (2021) Pathway-based analysis of anthocyanin diversity in diploid potato. PLoS ONE 16(4):e0250861. 10.1371/journal.pone.0250861 33914830 10.1371/journal.pone.0250861 PMC8084248

[CR200] Percival-Alwyn L, Barnes I, Clark MD, Cockram J, Coffey MP, Jones S, Kersey PJ, Kidner CA, Kosiol C, Li B, Marsh WA, Zhou J, Caccamo M, Milne I (2024) UKCropDiversity‐HPC: A collaborative high-performance computing resource approach for sustainable agriculture and biodiversity conservation. Plants People Planet. 10.1002/ppp3.10607

[CR48] Peterson R, Slovin JP, Chen C (2010) A simplified method for differential staining of aborted and non-aborted pollen grains. Int J Plant Biol 1(2):e13. 10.4081/pb.2010.e13

[CR49] Pham GM, Hamilton JP, Wood JC, Burke JT, Zhao H et al (2020) Construction of a chromosome-scale long-read reference genome assembly for potato. GigaScience. 10.1093/gigascience/giaa100 32964225 10.1093/gigascience/giaa100 PMC7509475

[CR50] Poland JA, Brown PJ, Sorrells ME, Jannink JL (2012) Development of high-density genetic maps for barley and wheat using a novel two-enzyme genotyping-by-sequencing approach. PLoS ONE 7:e32253. 10.1371/journal.pone.0032253 22389690 10.1371/journal.pone.0032253 PMC3289635

[CR51] Potato Genome Sequencing Consortium (2011) Genome sequence and analysis of the tuber crop potato. Nature 475:189–195. 10.1038/nature10158 21743474 10.1038/nature10158

[CR52] Rosyara UR, De Jong WS, Douches DS, Endelman JB (2016) Software for genome-wide association studies in autopolyploids and its application to potato. Plant Genome 9:1–10. 10.3835/plantgenome2015.08.0073 10.3835/plantgenome2015.08.0073 27898814

[CR53] Sato H, Koizumi R, Itoyama R, Ichisawa M, Negishi J, Sakuma R, Furusho T, Sagane Y, Takano K (2019) Free amino acids in potato (*Solanum tuberosum*) may cause egumi-taste in food products. Potato Res 62:305–314. 10.1007/s11540-019-9412-9

[CR54] Sharma SK, MacKenzie K, McLean K, Dale F, Daniels S et al (2018) Linkage disequilibrium and evaluation of genome-wide association mapping models in tetraploid potato. G3-Genes Genomes Genet. 10.1534/g3.118.200377 10.1534/g3.118.200377 PMC6169395 30082329

[CR55] Sharma SK, McLean K, Hedley PE, Dale F, Daniels S, Bryan GJ (2024) Genotyping-by-sequencing targets genic regions and improves resolution of genome-wide association studies in autotetraploid potato. Theor Appl Genet 137:180. 10.1007/s00122-024-04651-8 38980417 10.1007/s00122-024-04651-8 PMC11233353

[CR56] Śliwka J, Wasilewicz-Flis I, Jakuczun H, Gebhardt C (2008) Tagging quantitative trait loci for dormancy, tuber shape, regularity of tuber shape, eye depth and flesh colour in diploid potato originated from six *Solanum* species. Plant Breeding 127(1):49–55. 10.1111/j.1439-0523.2008.01420.x

[CR57] Śliwka J, Sołtys-Kalina D, Szajko K, Wasilewicz-Flis I, Strzelczyk-Żyta D, Zimnoch-Guzowska E, Jakuczun H, Marczewski W (2016) Mapping of quantitative trait loci for tuber starch and leaf sucrose contents in diploid potato. Theor Appl Genet 129(1):131–140. 10.1007/s00122-015-2615-9 26467474 10.1007/s00122-015-2615-9 PMC4703618

[CR58] Solms J (1971) Nonvolatile compounds and flavor. In: Ohloff G, Thomas AF (eds) Gustation and olfaction. Academic Press, London, UK, pp 92–110

[CR59] Sołtys-Kalina D, Szajko K, Stefańczyk E, Smyda-Dajmund P, Śliwka J, Marczewski W (2020) eQTL mapping of the 12S globulin cruciferin gene *PGCRURSE5* as a novel candidate associated with starch content in potato tubers. Sci Rep 10:17168. 10.1038/s41598-020-74285-5 33051578 10.1038/s41598-020-74285-5 PMC7553954

[CR60] Strzelczyk-Żyta D, Jakuczun H, Zimnoch-Guzowska E (1997) Zdolność do tworzenia męskich gamet *2n* w diploidalnych klonach ziemniaka z programu syntezy diploidalnych form rodzicielskich. Biuletyn Instytutu Ziemniaka 48:99–105

[CR61] Taylor MA, McDougall J, Stewart D (2007) Potato flavour and texture. In: Vreugdenhil D, Bradshaw JE, Gebhardt C, Govers F, Mackerron DKL, Taylor MA, Ross HA (eds) Potato biology and biotechnology. Advances and perspectives. Elsevier Science B.V, Amsterdam, pp 525–540

[CR62] Van Eck HJ (2007) Genetics of morphological and tuber traits. In: Vreugdenhil D, Bradshaw JE, Gebhardt C, Govers F, Mackerron DKL, Taylor MA, Ross HA (eds) Potato biology and biotechnology. Advances and perspectives. Elsevier Science B.V, Amsterdam, pp 91–115

[CR63] Van Eck HJ, Jacobs JME, van Dijk J, Stiekema WJ, Jacobsen E (1993) Identification and mapping of three flower colour loci of potato (*S. tuberosum* L.) by RFLP analysis. Theor Appl Genet 86:295–300. 10.1007/BF00222091 24193472 10.1007/BF00222091

[CR64] Van Eck HJ, Jacobs JME, Stam P, Ton J, Stiekema WJ, Jacobsen E (1994) Multiple alleles for tuber shape in diploid potato detected by qualitative and quantitative genetic analysis using RFLPs. Genetics 137:303–309. 10.1093/genetics/137.1.303 7914504 10.1093/genetics/137.1.303 PMC1205946

[CR65] Vos PG, Paulo MJ, Voorrips RE, Visser RGF, van Eck HJ et al (2017) Evaluation of LD decay and various LD-decay estimators in simulated and SNP-array data of tetraploid potato. Theor Appl Genet 130:123–135. 10.1007/s00122-016-2798-8 27699464 10.1007/s00122-016-2798-8 PMC5214954

[CR66] Wasilewicz-Flis I, Jakuczun H (2001) Estimation of pollen fertility in potato. Monografie i Rozprawy Naukowe IHAR, Radzików, Poland 10:121–122

[CR67] Zhang Y, Cheng S, De Jong D, Griffiths H, Halitschke R, De Jong W (2009) The potato *R* locus codes for dihydroflavonol 4-reductase. Theor Appl Genet 119:931–937. 10.1007/s00122-009-1100-8 19588118 10.1007/s00122-009-1100-8 PMC2729421

[CR68] Zhang C, Wang P, Tang D, Yang Z, Fei L, Qi J, Tawari NR, Shang Y, Li C, Huang S (2019) The genetic basis of inbreeding depression in potato. Nat Genet 51:374–378. 10.1038/s41588-018-0319-1 30643248 10.1038/s41588-018-0319-1

[CR69] Zia MAB, Demirel U, Nadeem MA, Çaliskan ME (2020) Genome-wide association study identifies various loci underlying agronomic and morphological traits in diversified potato panel. Physiol Mol Biol Plants 26(5):1003–1020. 10.1007/s12298-020-00785-3 32377049 10.1007/s12298-020-00785-3 PMC7196606

[CR70] Zimnoch-Guzowska E, Flis B (2021) Over 50 years of potato parental line breeding programme at the Plant Breeding and Acclimatization Institute in Poland. Pot Res 64:743–760. 10.1007/s11540-021-09503-2

