# Supplementary figures and images for: Historical data provide new insights into inheritance of traits important for diploid potato breeding
Source: Planta. 2025 Feb 27;261(4):69. doi: 10.1007/s00425-025-04618-z (PMC11868143; doi:10.1007/s00425-025-04618-z)

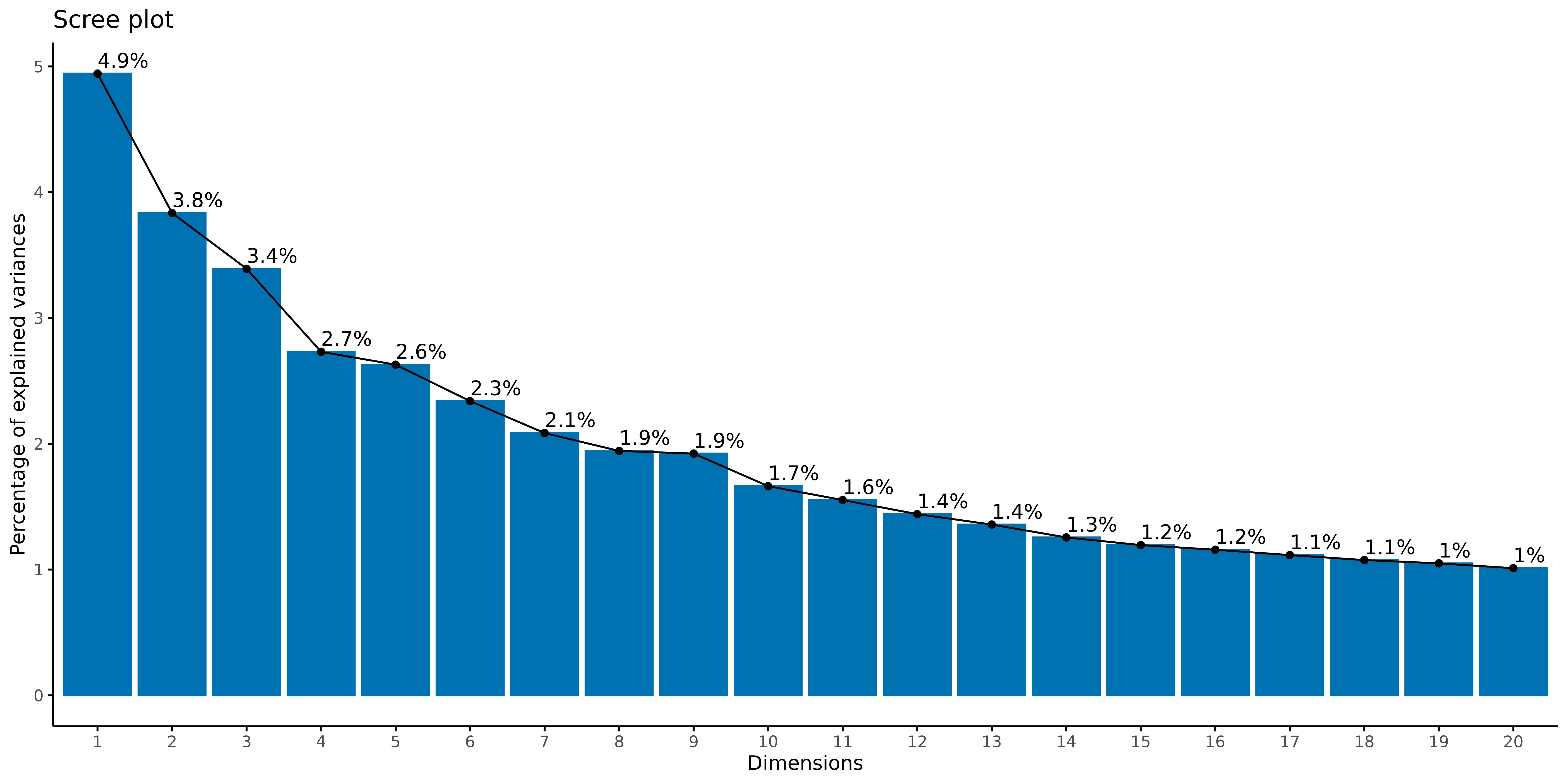

Supplement: Supplementary file 3 — Supplementary Fig. S3 Screeplot from the Principal Component Analysis (PCA) displaying the number of principal components versus their corresponding eigenvalues. PCA performed using 39,756 GBS SNPs (PNG 376 KB) [file 425_2025_4618_MOESM3_ESM.png]

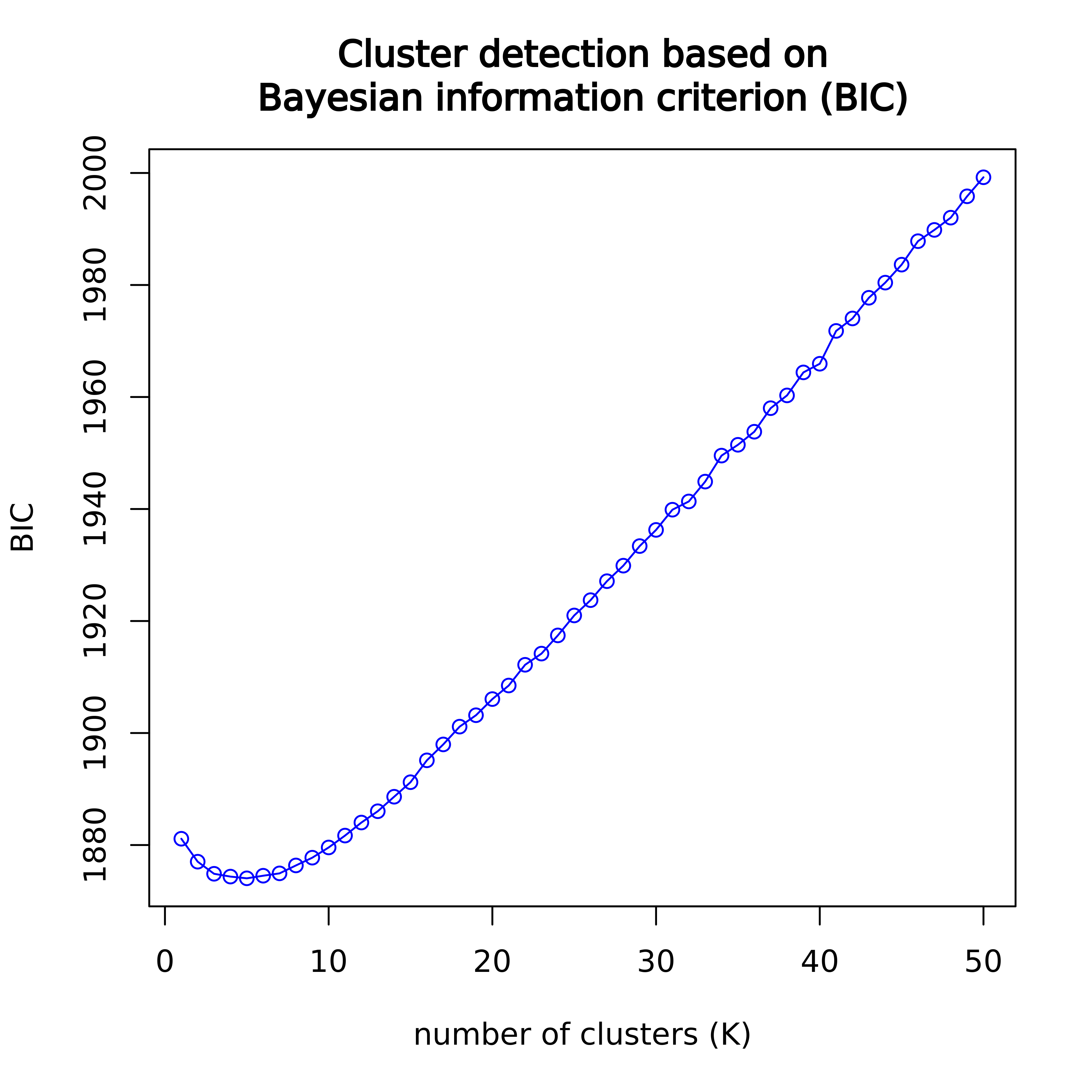

Supplement: Supplementary file 4 — Supplementary Fig. S4 Bayesian information criterion (BIC) statistical measure of goodness of fit curve for detecting optimal number of clusters (k-means) or subpopulations (Q) in DDP obtained using 39,756 GBS SNPs (PNG 301 KB) [file 425_2025_4618_MOESM4_ESM.png]
