# Supplementary material for: Historical data provide new insights into inheritance of traits important for diploid potato breeding
Source: Planta. 2025 Feb 27;261(4):69. doi: 10.1007/s00425-025-04618-z (PMC11868143; doi:10.1007/s00425-025-04618-z)
Supplement: Supplementary file 13 — The legends for the Supplementary files (20 KB) [file 425_2025_4618_MOESM13_ESM.docx]

**Supplementary material**

**Historical data provide new insights into inheritance of traits important for diploid potato breeding**

**Authors**

Jadwiga Śliwka^1^, Iwona Wasilewicz-Flis^1^, Henryka Jakuczun^1^, Marta Janiszewska^1^, Paulina Smyda-Dajmund^1^, Karen McLean^2^, Ewa Zimnoch-Guzowska^1^, Glenn J. Bryan^2^, Sanjeev Kumar Sharma^2^

**Affiliation**

^1^Plant Breeding and Acclimatization Institute - National Research Institute in Radzików, Młochów Division, Platanowa St. 19, Młochów, 05-831, Poland

^2^Cell and Molecular Sciences, The James Hutton Institute, Invergowrie, Dundee DD2 5DA, UK

**Supplementary Fig. S1** Overall distribution of (a) minor allele frequency (MAF), (b) maximum genotype frequency, and (c) polymorphism information content (PIC) for 39,756 GBS SNPs used in the study

**Supplementary Fig. S2** Chromosome-wise distribution of (a) minor allele frequency (MAF), (b) maximum genotype frequency, and (c) polymorphism information content (PIC) for 39,756 GBS SNPs used in the study. SNPs located in the chromosome 10 tuber shape and eye depth QTL region as reported by Sharma et al. (2024) are highlighted in red

**Supplementary Fig. S3** Screeplot from the Principal Component Analysis (PCA) displaying the number of principal components versus their corresponding eigenvalues. PCA performed using 39,756 GBS SNPs

**Supplementary Fig. S4** Bayesian information criterion (BIC) statistical measure of goodness of fit curve for detecting optimal number of clusters (k-means) or subpopulations (Q) in DDP obtained using 39,756 GBS SNPs

**Supplementary Fig. S5** Q-Q plots comparing the inflation of *p*-values for the four principal GWAS models deployed for each genetic (gene action) model for all 10 traits (YLD, Yield; MTW, Mean tuber weight; SHP, Tuber shape; REG, Regularity of tuber shape; EYE, Tuber eye depth; PUR, Purple tuber skin colour; TFC, Tuber flesh colour; TSC, Tuber starch content; TST, Boiled tuber taste; POL, Pollen fertility). Red circles: Naïve model; Green squares: K model; Blue diamonds: Q model; and Black triangles: QK model. Red line indicates *p*-values under the expected normal distribution

**Supplementary Fig. S6** Graphical illustration of the non-redundant set of unique QTL-MTAs (a, K models; b, QK models) listed in Table 5 (trait codes: YLD, Yield; MTW, Mean tuber weight; SHP, Tuber shape; REG, Regularity of tuber shape; EYE, Tuber eye depth; PUR, Purple tuber skin colour; TFC, Tuber flesh colour; TSC, Tuber starch content; TST, Boiled tuber taste; POL, Pollen fertility). Black dashed line depicts GWAS main significance threshold set to 1e-5 -log_10_(*p*) value while actual significance thresholds obtained separately for each trait MTA using Bonferroni-type multiple testing correction method “M.eff” (genome-wide α = 0.05) are provided in Table 5. The chromosome heatmaps below each plot illustrate SNP density (bin size = 1 Mb)

**Supplementary Tables**

**Supplementary Table S1** List of potato diploid diversity panel genotypes employed in the study including their population group (cluster) membership details and main breeding aims

**Supplementary Table S2** List of barcodes employed to construct 96-plex GBS libraries

**Supplementary Table S3** List of genes overlapping GBS SNPs including impact category count

**Supplementary Table S4** Genomic control inflation factor (λ_GC_) values for each trait and 'genetic model x GWAS model' combination. Trait codes: YLD, Yield; MTW, Mean tuber weight; SHP, Tuber shape; REG, Regularity of tuber shape; EYE, Tuber eye depth; PUR, Purple tuber skin colour; TFC, Tuber flesh colour; TSC, Tuber starch content; TST, Boiled tuber taste; POL, Pollen fertility

**Supplementary Table S5** List of significant QTL marker-trait associations (QTL-MTAs) detected in diploid diversity panel (DDP). QTL-MTAs appearing for all gene action models are included. Trait codes: YLD, Yield; MTW, Mean tuber weight; SHP, Tuber shape; REG, Regularity of tuber shape; EYE, Tuber eye depth; PUR, Purple tuber skin colour; TFC, Tuber flesh colour; TSC, Tuber starch content; TST, Boiled tuber taste; POL, Pollen fertility

**Supplementary Table S6** List of (a) top QTL-MTAs detected in tuber shape and tuber eye depth candidate GWAS peaks and (b) QTL-MTAs for these two traits common between DDP and Sharma et al. (2024). Cells highlighted in grey contain the most significant -log_10_(*p*) values corresponding to the respective gene action models listed under column 'Ranking Model'
